# Multifunctional *s*-triazine-BODIPY conjugates: synthetic strategies, photophysical insights, and emerging applications

**DOI:** 10.1039/d5ra04624f

**Published:** 2025-08-08

**Authors:** Laxmipriya Nayak, Subhadeep Acharya, Supriya Routray, Simran Pattnaik, Rashmirekha Satapathy

**Affiliations:** a Department of Chemistry, Ravenshaw University Cuttack-753003 Odisha India rashmi@ravenshawuniversity.ac.in

## Abstract

The integration of *s*-triazine and BODIPY scaffolds has emerged as a versatile strategy for developing multifunctional conjugates with tailored photophysical and biological properties. This review provides a detailed overview of the different design principles, synthetic strategies, and applications of *s*-triazine BODIPY conjugates in the last decade. Key photophysical parameters such as absorption maxima, fluorescence lifetimes, quantum yields, and singlet oxygen generation efficiencies are examined in the context of their structure–property relationships. The diverse applications of the conjugates are categorized into three primary domains such as biological application, including imaging and photodynamic therapy; sensing applications with discussions about mechanisms like PET, ICT, ESIPT and FRET; and advanced material applications, including their use as molecular rotors, liquid crystals, photocatalysts for CO_2_ reduction, and components in solar cells and optoelectronic devices. This work underscores the growing importance of *s*-triazine-BODIPY conjugates as a modular platform for future innovations across materials science, analytical chemistry, and biomedical fields.

## Abstract 2

The integration of *s*-triazine and BODIPY scaffolds has emerged as a versatile strategy for developing multifunctional conjugates with tailored photophysical and biological properties.

## Graphical abstract

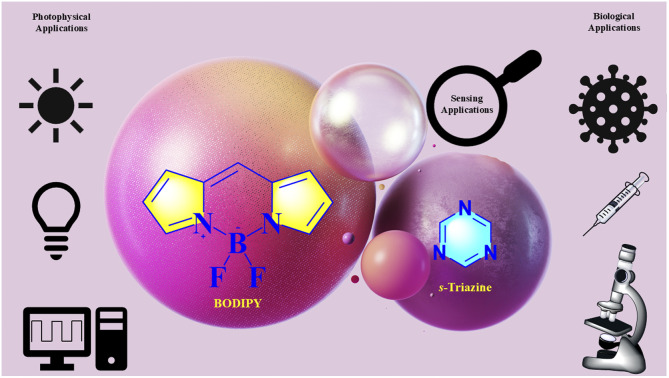

## 1. Introduction

### 1.1. Introduction to triazine

**Fig. 1:**
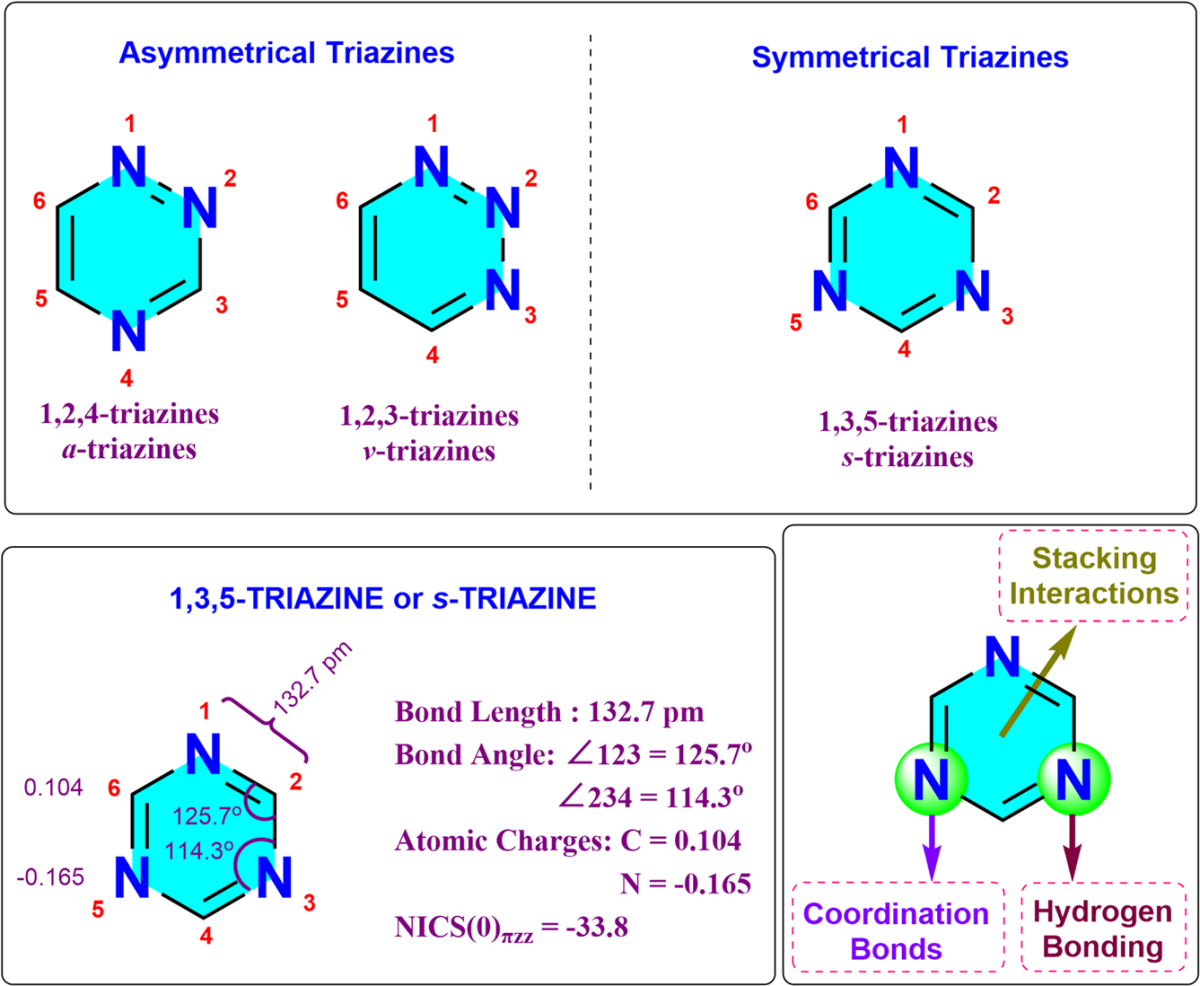
Structure and properties of triazines.

Triazines are a significant group of aromatic heterocyclic compounds. These molecules are characterized by a six-membered ring structure incorporating three nitrogen atoms. They exist in three distinct regioisomeric forms: 1,2,3-triazines (*ν*-triazines), 1,2,4-triazines (*α*-triazines), and 1,3,5-triazines (*s*-triazines) (Fig. 1a).^1–4^*s*-Triazine is a symmetrical heterocyclic compound that serves as a foundation for the construction of diverse and multifunctional molecular architectures.^5–7^ It exhibits a nearly planar structure and displays aromaticity comparable to benzene (Fig. 1b).^8–10^ This aromaticity is supported by nucleus-independent chemical shift (NICS(0)_πzz_) calculations, which yield a value of −33.8 for 1,3,5-triazine, closely resembling the value of −36.1 for benzene.^11^ The polarization of the carbon-nitrogen bonds (C=N) within the s-triazine ring results in a significant accumulation of electron density on the nitrogen atoms. This is quantified by Hirshfeld charge analysis, which assigns a partial positive charge of +0.104 to the carbon atoms and a partial negative charge of −0.165 to the more electronegative nitrogen atoms (Fig. 1b). Despite possessing two distinct bond angles (∠123 = 125.7° and ∠234 = 114.3°), the identical bond lengths within the 1,3,5-triazine ring (132.7 pm) contribute to its unique symmetry.

#### 1.1.1. Various synthetic approaches for the synthesis of *s*-triazine core

**Fig. 2:**
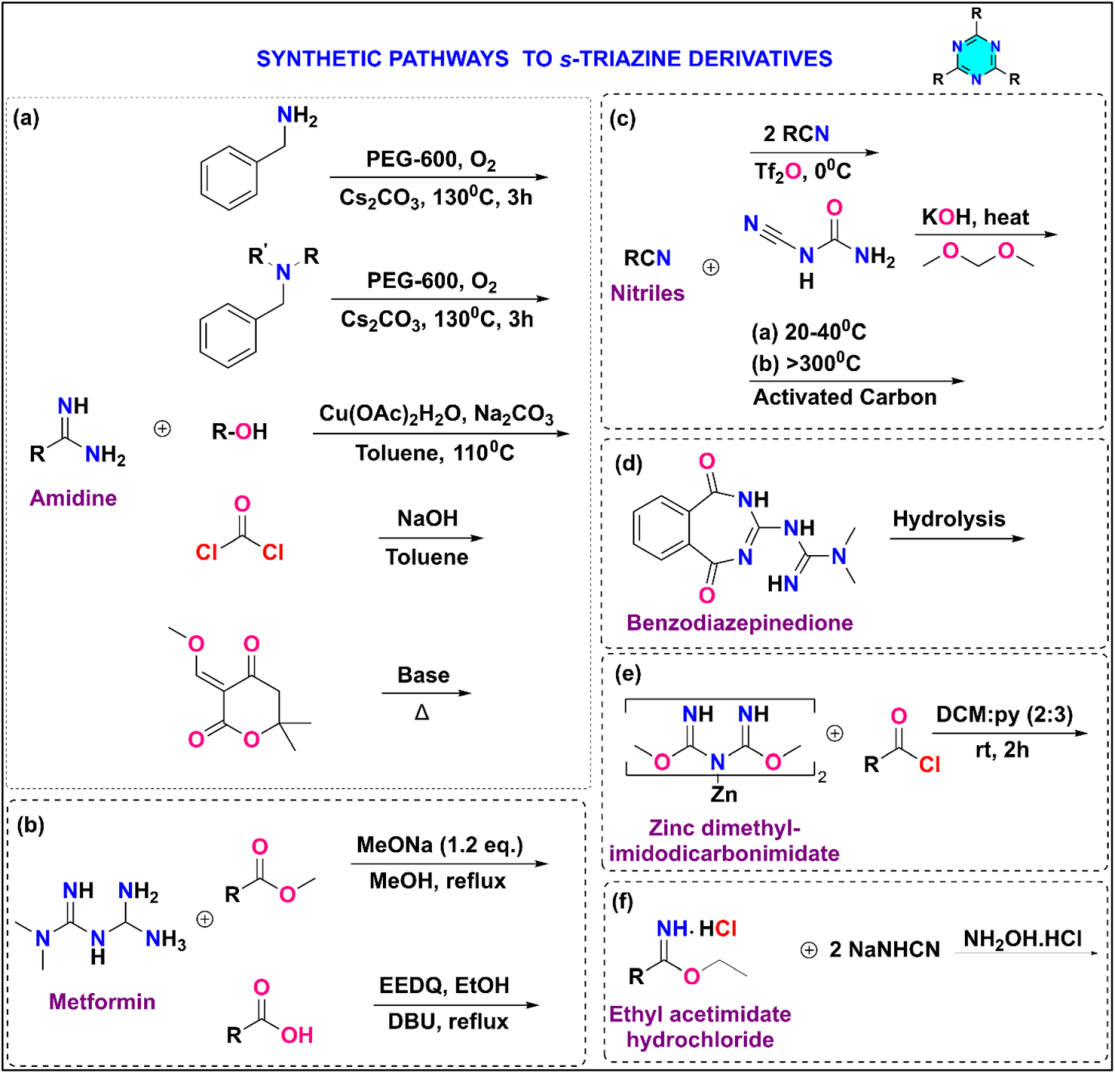
Synthetic pathways to *s*-triazine derivatives.

A diverse array of synthetic strategies have been employed for the preparation of *s*-triazine derivatives, capitalizing on readily available and inexpensive starting materials (Fig. 2). For example, amidines (Fig. 2a) have been utilized as precursors for *s*-triazine synthesis through various approaches such as metal-free coupling with primary amines,^12^ copper-catalyzed coupling with tertiary amines,^12^ chemoselective and base-free aerobic oxidation with alcohols,^13–16^ reaction with phosgene gas,^17^ and condensation with diones.^18^ Substituted *s*-triazines can also be synthesized from metformin through reactions with either esters or acids (Fig. 2b).^19,20^ Additionally, metformin, when reacted with primary alcohols using graphene oxide as a catalyst, yields substituted s-triazines.^21^ Lewis acid-catalyzed cyclization of nitriles provides access to substituted symmetrical triazines.^22^ Additionally, 1,3,5-triazine can be synthesized in a single step by trimerizing nitriles (Fig. 2c).^23^ Researchers have documented the formation of substituted *s*-triazines by employing cycloaddition reactions of diisocyanates (DICY) with aryl and aliphatic nitriles.^24^ In industrial settings, *s*-triazine derivatives are frequently synthesized starting from hydrogen cyanide.^25^ 1,3,5-triazine derivatives can be synthesized by subjecting benzodiazepinediones to rearrangement reactions, such as hydrolysis or alcoholysis (Fig. 2d).^26^ Furthermore, activated derivatives of carboxylic acid can be reacted with zinc dimethyl imidodicarbonimidic to furnish *s*-triazine derivatives (Fig. 2e).^27^ Additionally, ethyl acetimidate hydrochloride, upon reaction with two equivalents of sodium cyanamide (NaNHCN) followed by treatment with hydroxylamine hydrochloride, affords *s*-triazine derivatives (Fig. 2f).^28^

**Fig. 3:**
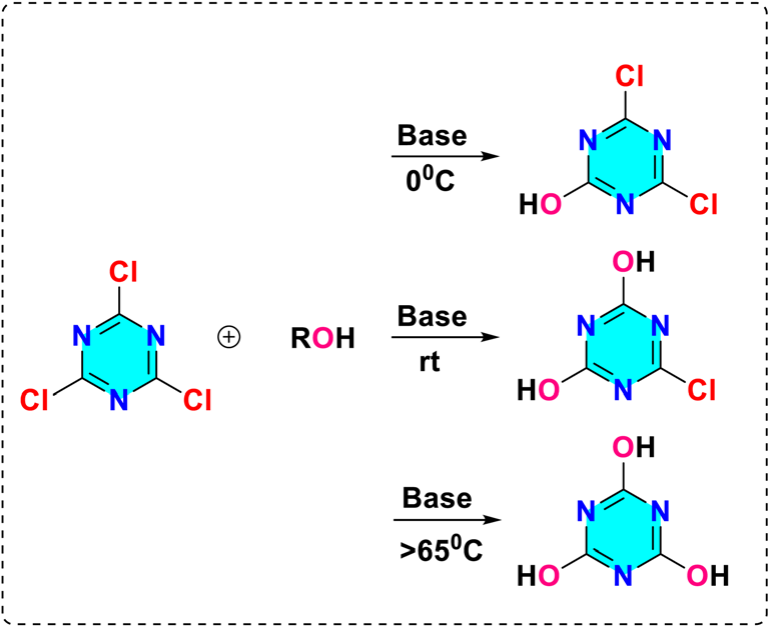
Temperature-dependent aromatic nucleophilic substitution of cyanuric chloride.

Beyond these direct synthetic approaches, a highly versatile, inexpensive, and commercially accessible starting material for the synthesis of *s*-triazine derivatives is cyanuric chloride (2,4,6-trichloro-1,3,5-triazine).^29^ It demonstrates the differential reactivity dependent on temperature for the displacement of the chloro group by nucleophiles, in conjunction with aromatic nucleophilic substitution (Fig. 3). Typically, the first chloro group substitution takes place at freezing temperature (0 °C). The second chloro group substitution occurs at room temperature, while the third substitution happens at higher temperatures, depending on the reactivity of the selected nucleophile (70–100 °C). The versatility of the 1,3,5-triazine core in substitutions enables synthetic flexibility, allowing for the creation of a variety of derivatives for different applications.

### 1.2. Introduction to BODIPY

**Fig. 4:**
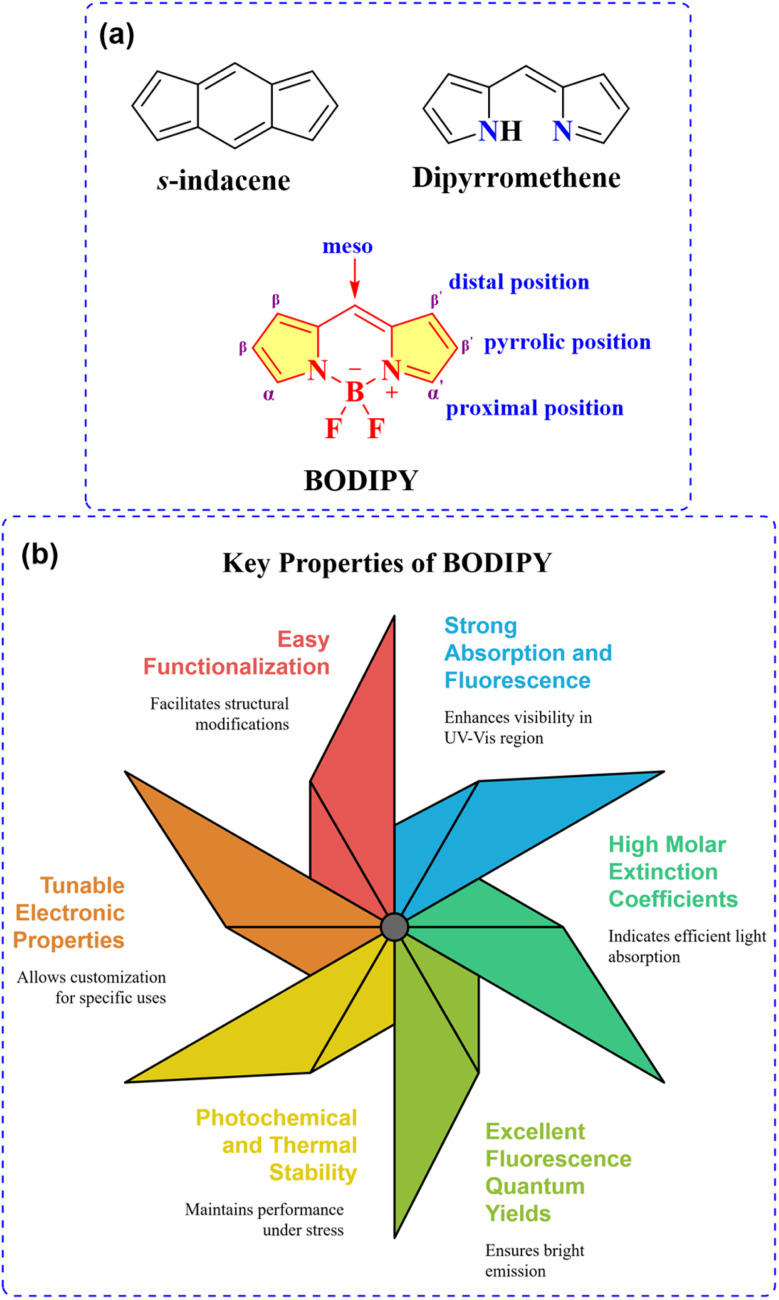
(a) Structure of *s*-indacene, dipyrromethene, and BODIPY; (b) key characteristics of BODIPY.

Within the expansive realm of highly fluorescent dyes, those derived from the 4,4-difluoro-4-bora-3*a*,4*a*-diaza-*s*-indacene scaffold (commonly referred to as BODIPY dyes) stand out as particularly promising, having experienced a remarkable surge in popularity within the scientific community (Fig. 4).^30,31^ In 1968, Kreuzer and Treibs first described the BODIPY-based fluorescent dyes,^32^ however, it wasn't until the early 1990s that these dyes garnered significant attention, largely attributed to the pioneering work of Boyer and colleagues, who demonstrated their exceptional suitability as active media for tunable lasers.^33,34^ BODIPY dyes belong to the class of cyclic cyanines, characterized by a chromophoric core comprising a dipyrrin (or dipyrromethene) unit with a conjugated π-system chelated by a difluoroboron bridge. The dipyrrin moiety serves as the primary contributor to the electronic transitions within the molecule. Crucially, the coordinated BF_2_ unit plays a pivotal role by restricting conformational flexibility, thereby maintaining a planar molecular geometry. This minimizes energy loss through non-radiative decay pathways like internal conversion and enhances emission efficiency.^31,35^

#### 1.2.1. Synthesis of the BODIPY core

**Scheme 1:**
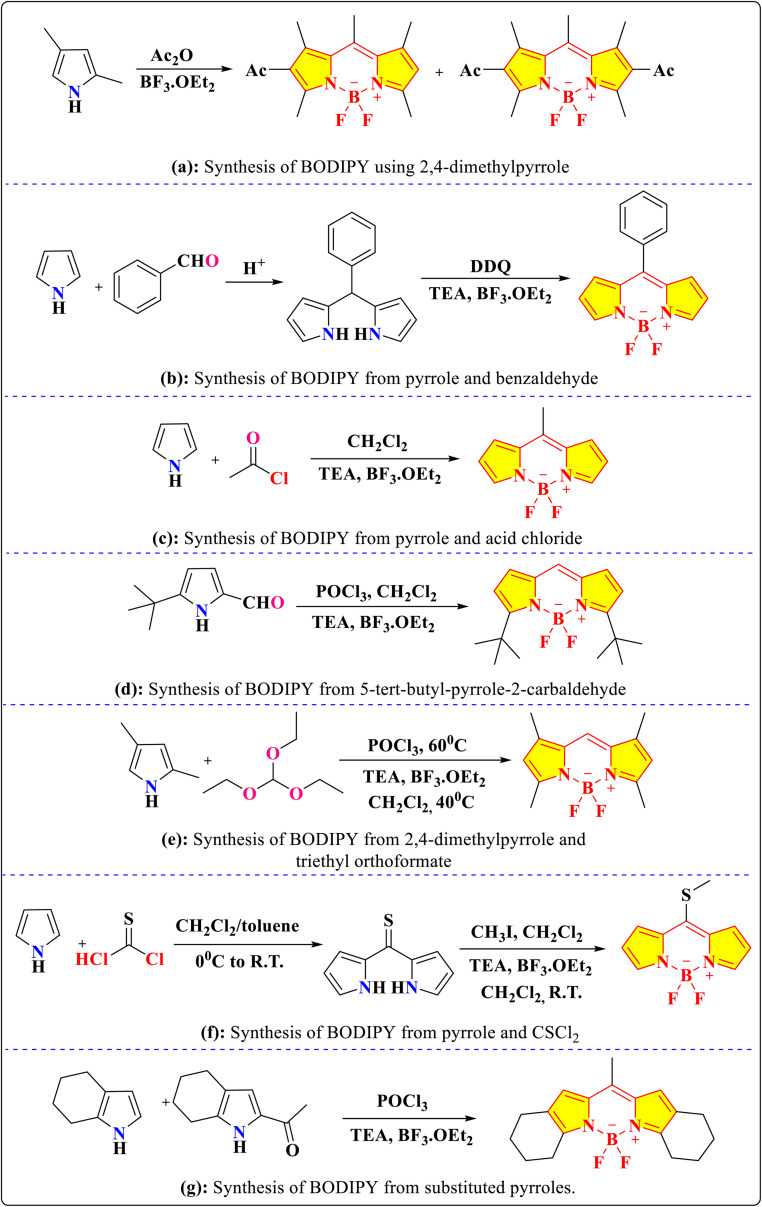
Various methods for the synthesis of BODIPY core.

The various approaches for synthesizing the BODIPY core are detailed in the following sections. In 1968, Treibs and Kreuzer unexpectedly discovered the remarkably fluorescent F-BODIPY framework while acylating 2,4-dimethylpyrrole using BF_3_·OEt_2_ and acetic anhydride. They isolated the vibrantly coloured di- and mono-substituted BODIPYs in less than 10% yields. The interaction between dipyrrin and BF_3_ encourages the tetrahedral arrangement at the boron centre (Scheme 1a).^32^

Another approach involves the acid-catalyzed condensation of an aldehyde with pyrrole, followed by oxidation with DDQ and reaction with BF_3_·OEt_2_, affording the target fluorophore with yields of around 22% and an absorption maximum (*λ*_abs_) at 503 nm in toluene (Scheme 1b).^36^ An alternative one-pot synthesis utilizes the interaction of pyrrole with an acid chloride, subsequently treated with BF_3_·OEt_2_, to produce the BODIPY core in 21% yield (Scheme 1c).^33,37^ This method yields a dye with an absorption maximum (*λ*_abs_) of 494 nm, an emission maximum (*λ*_em_) of 512 nm, and a high quantum yield (*Φ*_F_) of 0.87 in dichloromethane.

Furthermore, BODIPY derivatives can be obtained through the self-condensation of substituted pyrrole-2-carbaldehydes using POCl_3_, followed by the addition of triethylamine (TEA) and BF_3_·OEt_2_, achieving a lower yield of 15% (Scheme 1d).^38^ Another strategy employs the reaction of 2,4-dimethylpyrrole with triethyl orthoformate and POCl_3_, followed by boron complexation, which has been shown to produce the desired product in a 40% yield (Scheme 1e).^39^

More recent developments include the use of thiophosgene as a one-carbon linker between two pyrrole units, which, after subsequent methylation and reaction with BF_3_·OEt_2_ yields the BODIPY core in 26% yield, exhibiting a *λ*_abs_ at 485 nm and a *λ*_em_ at 525 nm (Scheme 1f).^40^ Additionally, the synthesis of BODIPYs with fused ring systems, such as those incorporating cyclohexane moieties, has been achieved from appropriately substituted pyrroles. These architecturally rigid derivatives exhibit red-shifted absorption and emission spectra, with *λ*_abs_ in the range of 534–543 nm and *λ*_em_ between 543–551 nm, along with high quantum yields of 0.76–0.89 (Scheme 1g).^41^

#### 1.2.2. Functionalization of BODIPY core

**Fig. 5:**
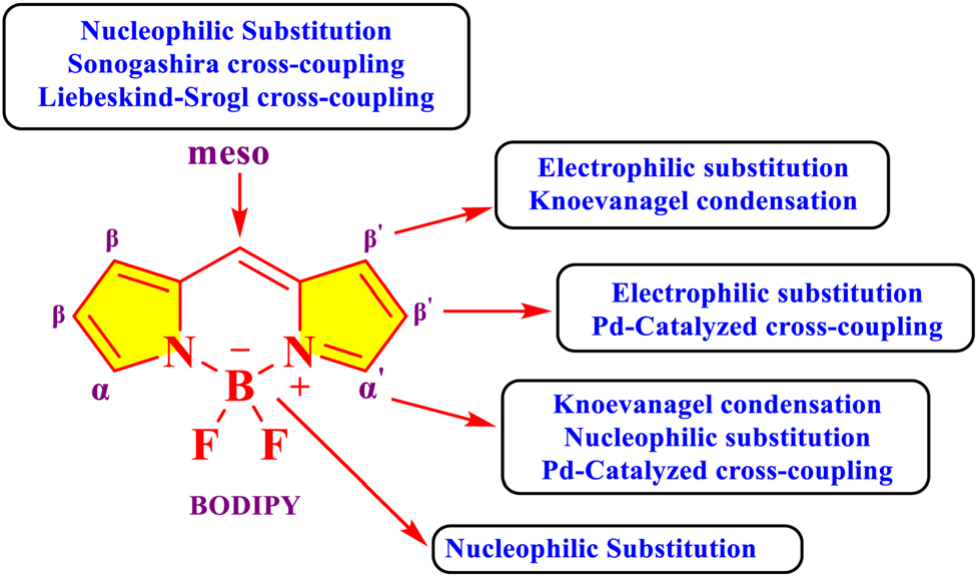
Different functionalization positions of BODIPY core.

**Scheme 2:**
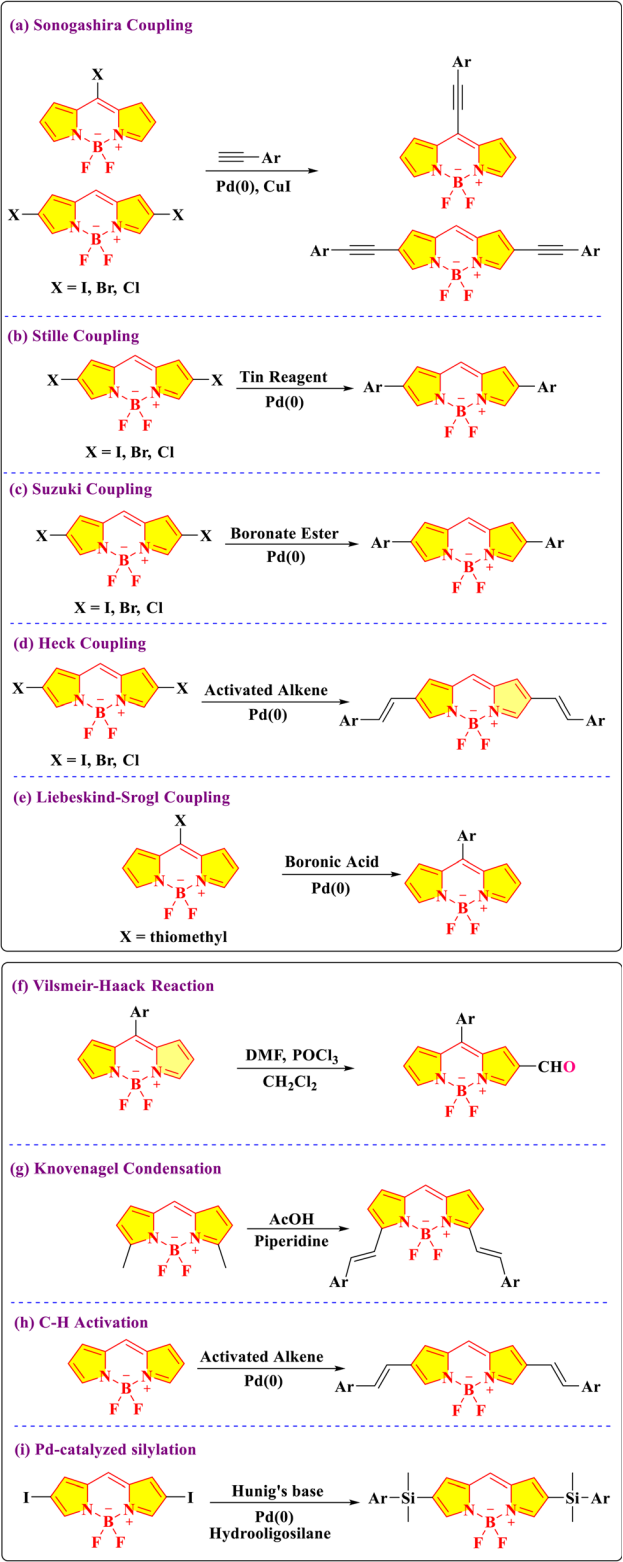
Different methods for the functionalization of the BODIPY core.

The functionalization of BODIPY (boron-dipyrromethene) dyes is a critical area of research aimed at tailoring their photophysical and chemical properties for diverse applications (Fig. 5).^42^ This involves modifications at specific positions to fine-tune its properties, enabling its use in specialized applications. The meso position of the BODIPY core is frequently targeted for functionalization. Electron-donating or electron-withdrawing groups located at the meso-position possess the capacity to modulate the photophysical behavior, thus rendering this site particularly advantageous for the design of probes exhibiting tunable fluorescence responses.^43^ The meso-position of BODIPY participates in nucleophilic substitution, the Sonogashira cross-coupling reaction (Scheme 2a), and the Liebeskind–Srogl cross-coupling reaction (Scheme 2e).^43^

The alpha-positions of the BODIPY core are also highly reactive and frequently modified. Halogenation at these positions enhances intersystem crossing, enabling the development of photosensitizers for photodynamic therapy.^44^ These halogenated derivatives are also useful precursors for aromatic nucleophilic substitution (S_N_Ar) and coupling reactions, such as Suzuki (Scheme 2c), Heck (Scheme 2d), and Sonogashira couplings (Scheme 2a). Such reactions introduce aryl, alkyl, or alkyne groups, providing opportunities to design BODIPY dyes with extended conjugation systems that exhibit red-shifted absorption and emission spectra.^45,46^

Functionalization at the beta-positions of the BODIPY core has been explored to further expand its electronic conjugation. Substituents at these positions frequently induce bathochromic shifts in both the absorption and emission spectra, rendering the dye appropriate for near-infrared (NIR) applications. Additionally, steric hindrance introduced at these positions can reduce dye aggregation, improving their photostability and solubility.

The incorporation of functional groups at the boron center of BODIPY offers another strategy for modification. Substitution of the fluorine atoms with other ligands, such as alkoxy or aryloxy groups, can influence the dye's solubility, stability, and electronic properties.^47^ Similarly, incorporating chelating groups at the boron center allows the BODIPY dye to serve as a sensor molecule for the selective detection of metal ions or other analytes.

BODIPY dyes can also undergo post-synthetic modifications, including Knoevenagel condensations at the 3,5-methyl groups.^48^ This reaction introduces styryl or other conjugated systems, resulting in derivatives with red-shifted spectra and increased photostability. Additionally, aza-BODIPY dyes, where the meso-carbon is replaced with a nitrogen atom, exhibit significantly red-shifted absorption and emission, making them suitable for NIR imaging and photodynamic therapy.^49^ Overall, the versatility of BODIPY functionalization lies in its synthetic accessibility and modular structure. The ability to introduce a wide range of substituents at various positions allows researchers to design dyes with specific properties tailored to their applications, including high brightness, water solubility, biocompatibility, and tailored absorption/emission profiles. This adaptability continues to drive innovation in the use of BODIPY dyes across scientific and technological domains.

## 2. Application of triazine-BODIPY conjugates

### 2.1 Biological application of triazine-BODIPY conjugates

**Fig. 6:**
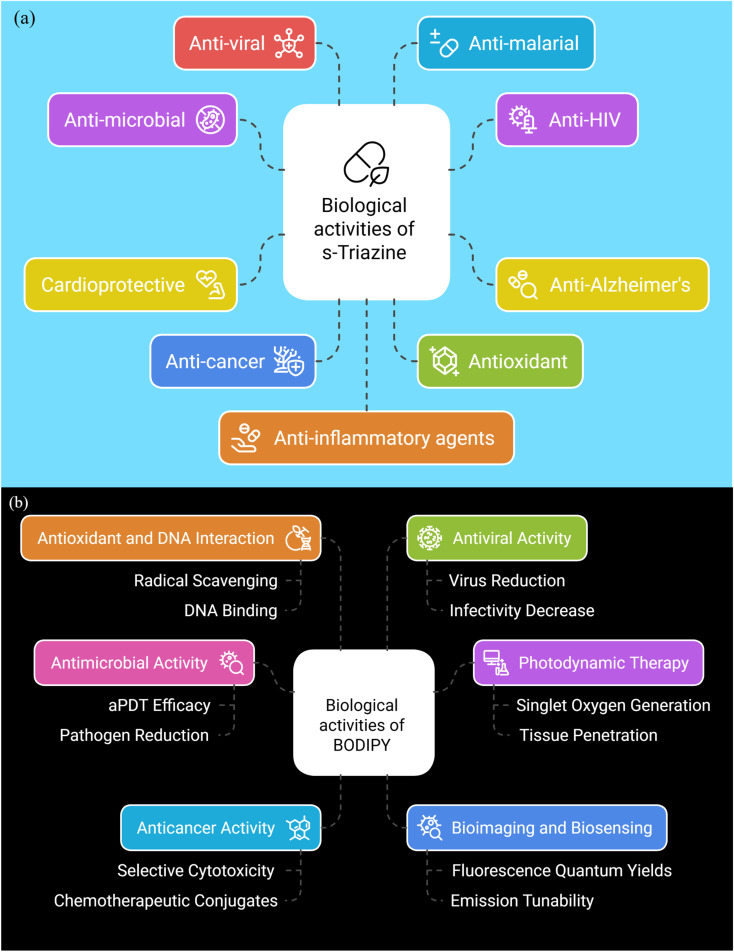
Biological activities of (a) *s*-triazine scaffold and (b) BODIPY scaffold.

The *s*-triazine scaffold serves as a foundation for designing biologically relevant molecules with recognized applications, including antibacterial, anticancer, anti-HIV, anti-Alzheimer's, anti-diabetic, anti-malarial, anti-inflammatory, and antioxidant properties (Fig. 6a). Many *s*-triazine-based molecules are part of FDA-approved drugs, such as bimiralisib A^50^ (anti-breast cancer), gedatolisib B^51,52^ (anti-breast cancer), enasidenib C^53^ (antileukemia), altretamine D^54^ (anti-ovarian cancer), tretamine E^28^ (antitumor), azacitidine F^55^ (antineoplastic agent), *etc.*

In parallel, BODIPY scaffolds have also emerged as versatile tools for applications like drug administration,^56–58^ bioimaging,^59–68^ artificial photosynthetic systems,^69–71^ and fluorescence sensing.^72–79^ In addition to their diagnostic applications, BODIPY derivatives have demonstrated potential as therapeutic agents, especially within the realm of photodynamic therapy (PDT)^80–82^ (Fig. 6b). Collectively, the distinct yet complementary biological and photochemical profiles of *s*-triazine and BODIPY provide a robust foundation for exploring their conjugates, which aim to synergize therapeutic efficacy with diagnostic precision.

**Scheme 3:**
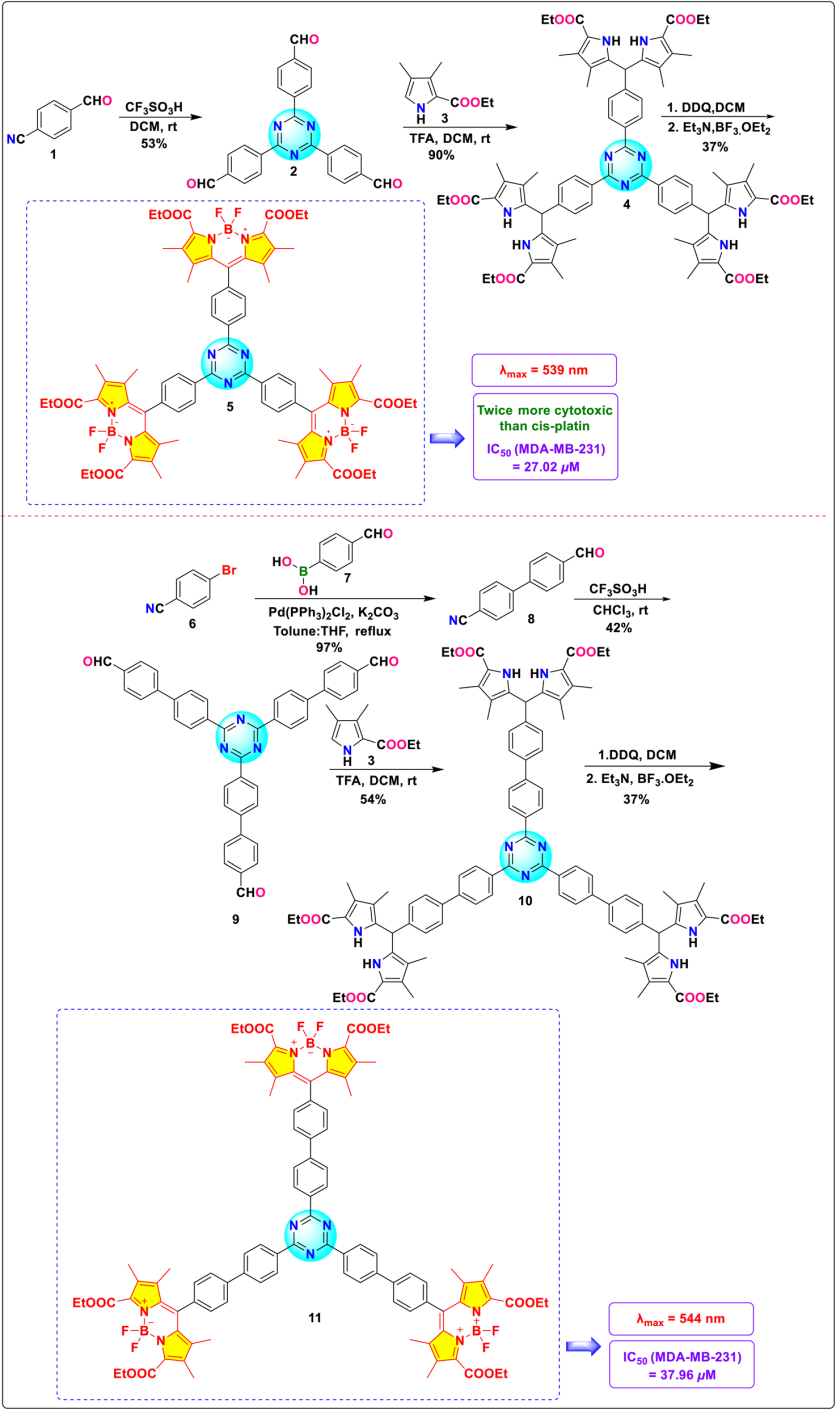
Synthetic route for *s*-triazine-BODIPY conjugates 5 and 11.

**Fig. 7:**
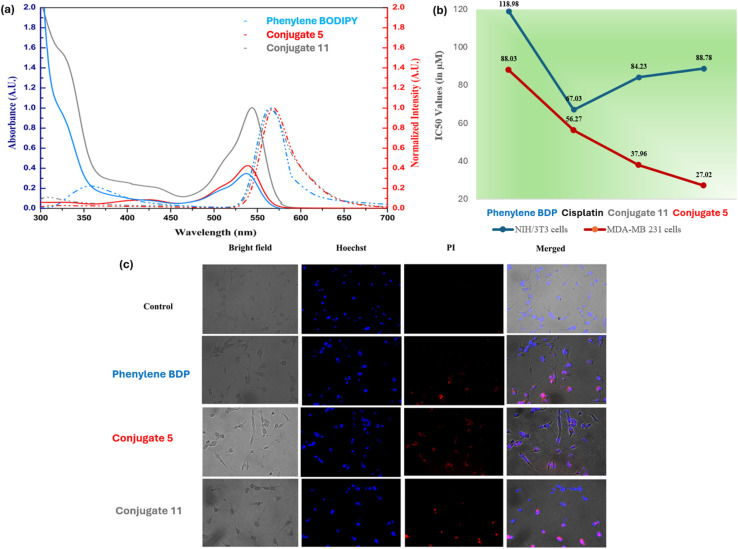
(a) Emission and absorption spectra of conjugates 5 and 11; (b) IC_50_ values of conjugates 5 and 11; (c) live-dead imaging of conjugates 5 and 11 using dyes Hoechst and PI. Reproduced with permission from ref. 75. Copyright 2024, Wiley-VCH.

Our group recently reported the synthesis and characterization of two novel *s*-triazine-BODIPY conjugates, 5 and 11 (Scheme 3).^83^ These compounds underwent a detailed analysis of their photophysical, computational, and *in vitro* anticancer properties, directly comparing them to a previously synthesized phenylene-cored analogue. Photophysical studies indicated a minor red shift in the absorption maxima of the *s*-triazine-BODIPY conjugates compared to the phenylene-based analogues, suggesting a subtle influence of the core structure on the electronic properties (Fig. 7). The anticancer properties of the conjugates were assessed *in vitro* through cytotoxicity assays on MDA-MB-231 (human breast adenocarcinoma cell line) and NIH/3T3 (mouse embryo fibroblast cell line). The results demonstrated a preferential cytotoxic effect of all BODIPY conjugates toward cancer cells, with minimal toxicity observed in normal cells. Conjugate 5 exhibited the highest cytotoxic potential, achieving an IC_50_ value of 27.02 μM, which is twice as potent as the established chemotherapeutic drug cisplatin (Fig. 7). LIVE/DEAD fluorescent images of cancer cells treated with conjugates using fluorescent dyes Hoechst 33 342 and Propidium Iodide (PI) demonstrated cell death at their IC_50_ value. These findings highlight the superior anticancer efficacy of the *s*-triazine-BODIPY conjugates in comparison to their phenylene-cored counterparts.

**Scheme 4:**
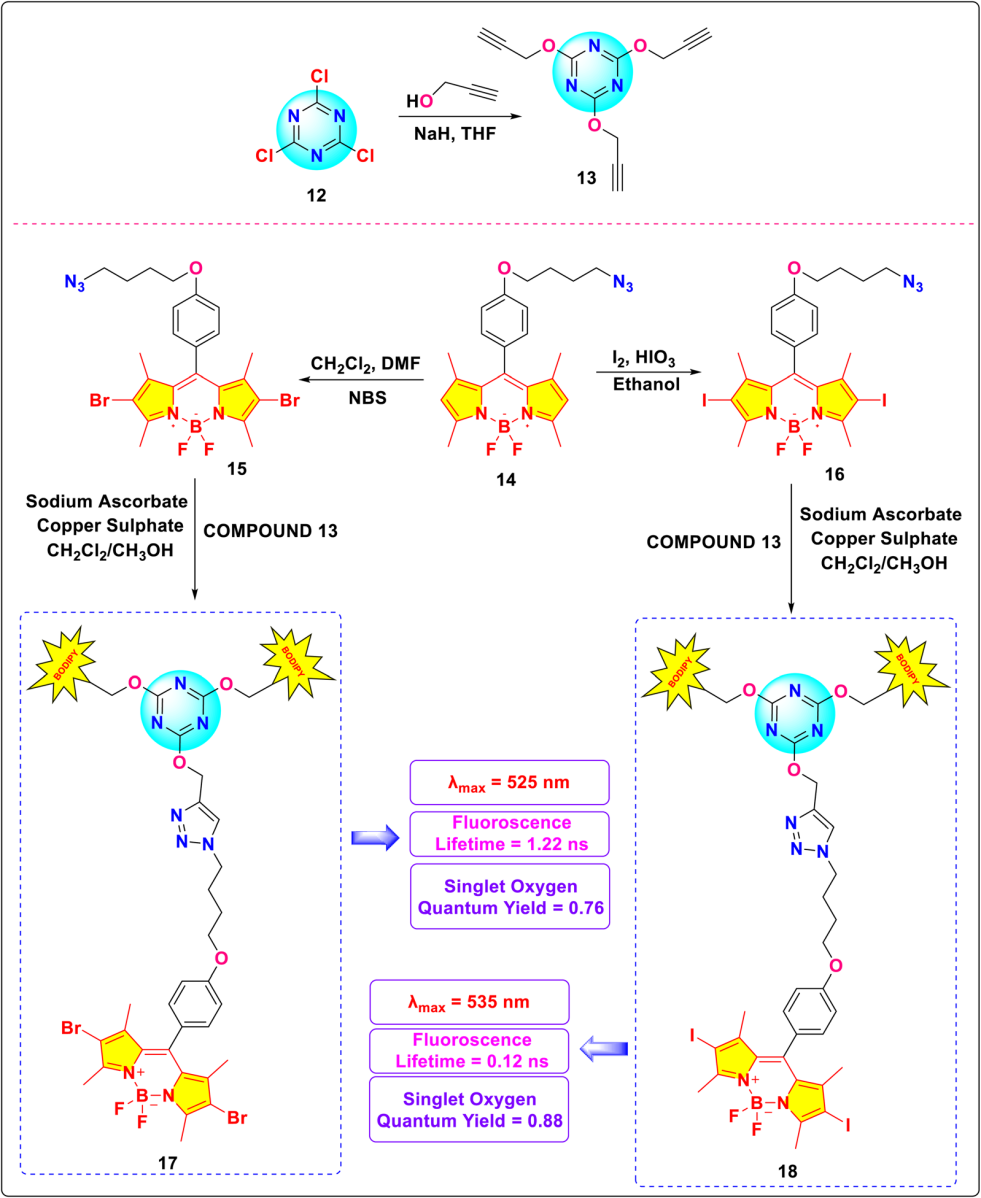
Synthetic route for *s*-triazine-BODIPY conjugates 17 and 18.

**Fig. 8:**
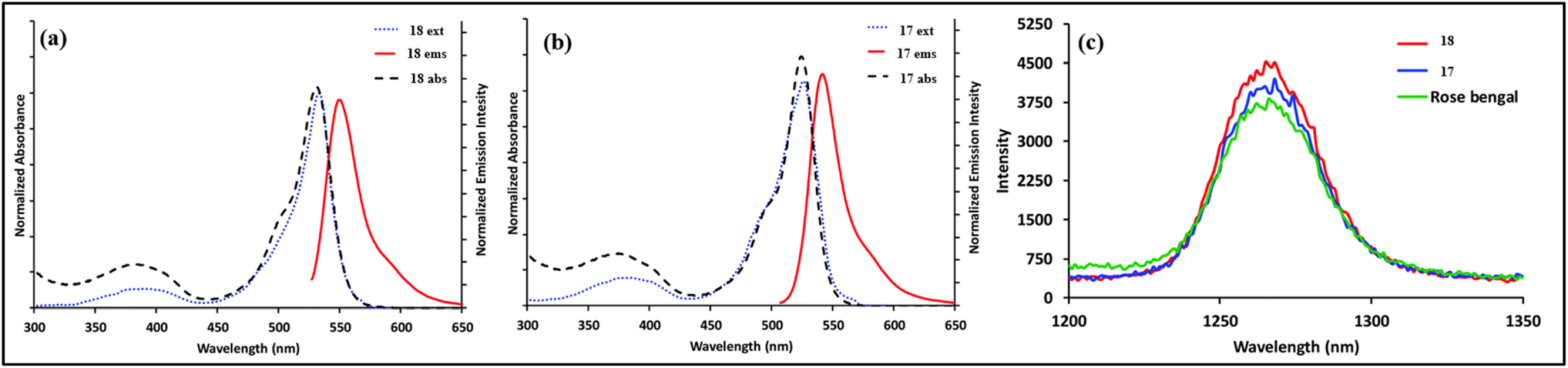
Emission and absorption spectra of conjugate (a) 18, (b) 17 in ethanol; (c) singlet oxygen phosphorescence with sensitization from conjugates 17, 18, and Rose Bengal in ethanol. Reproduced with permission from ref. 76. Copyright 2021, Royal Society of Chemistry.

Eçik and colleagues synthesized two halogenated *s*-triazine-BODIPY conjugates 17 (Brominated) and 18 (Iodinated), and studied their photodynamic therapy (PDT) properties by measuring their ability to generate singlet oxygen (Scheme 4).^84^ Conjugate 17 and 18 exhibited maximum absorption (*λ*_max_) at 525 nm and 535 nm, respectively (Fig. 8). The red shift (10 nm) in the case of the iodo-derivative was ascribed to the minimal influence of halogen atomic radius on the molecular π-electron system. Conjugate 17 exhibited a fluorescence lifetime of 1.22 ns due to increased intersystem crossing (ISC), while conjugate 18 displayed a non-fluorescent profile with a lifetime of 0.12 ns. The singlet oxygen quantum yields for conjugates 17 and 18 were 0.76 and 0.88, respectively. Subsequent biological assays confirmed cellular internalization of both conjugates with light-dependent cytotoxicity, supporting their potential efficacy in *in vitro* PDT.

**Fig. 9:**
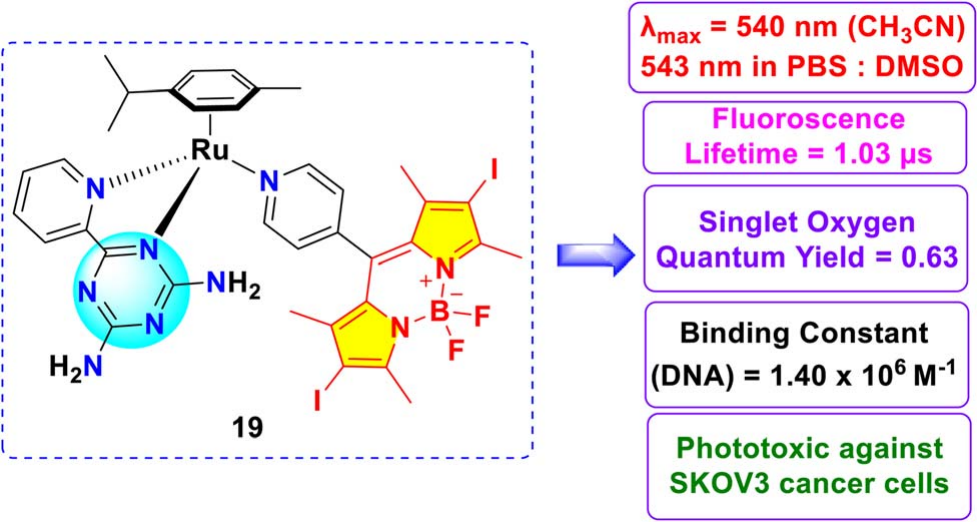
Structure of *s*-triazine-BODIPY conjugate 19.

Wang *et al.* described the synthesis of a novel ruthenium complex of *s*-triazine-BODIPY conjugates 19 (Fig. 9). Conjugate 19 exhibited absorption maxima (*λ*_max_) at 540 nm in acetonitrile and 543 nm in phosphate-buffered saline (PBS) : DMSO (8 : 1). The emission maxima (*λ*_em_) were observed at 568 nm in acetonitrile and 573 nm in PBS : DMSO (8 : 1). The cytotoxicity of the conjugate was investigated against human ovarian adenocarcinoma SKOV3 cells and the conjugate demonstrates ten times greater phototoxicity in presence of light irridiation than its dark toxicity. The conjugate also demonstrates the ability to generate singlet oxygen (^1^O_2_) with a quantum yield of 0.63, positioning it as a promising photosensitizer in photodynamic therapy (PDT). This remarkable PDT effectiveness is due to the synergistic combination of the iodinated BODIPY and Ru(ii) arene parts.

### 2.2 Sensing application of triazine-BODIPY conjugates

**Fig. 10:**
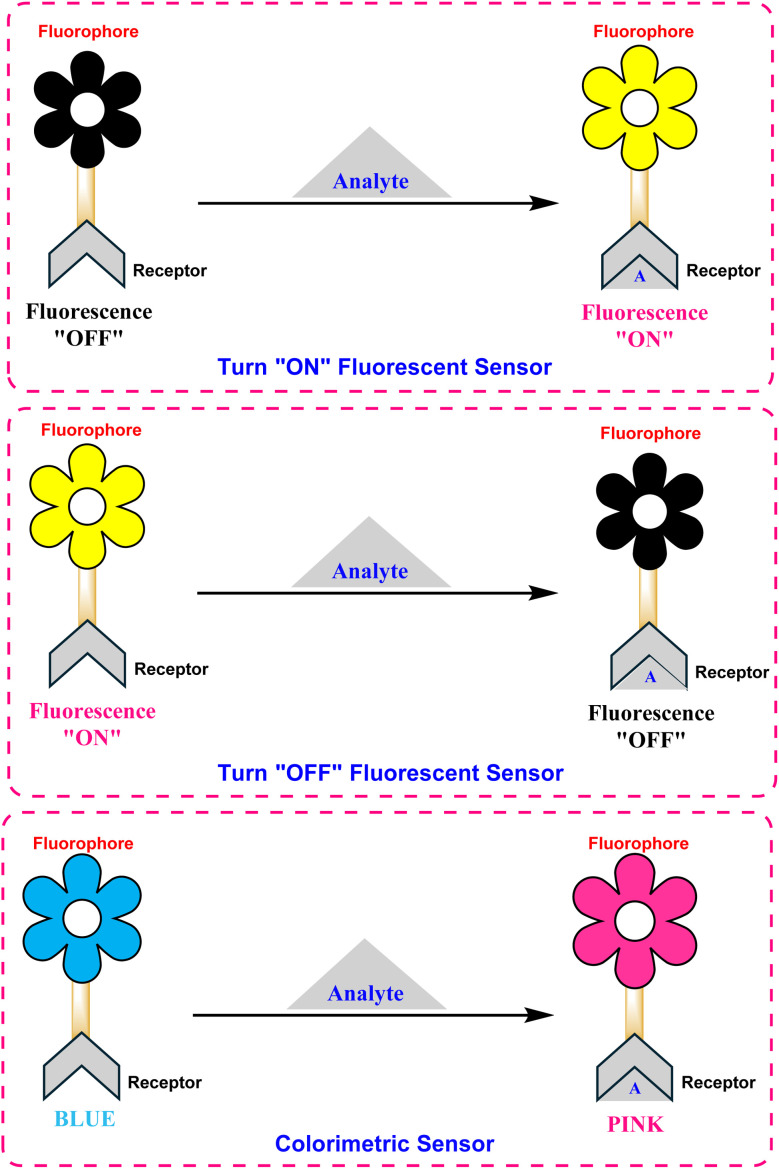
Schematic representation of different types of BODIPY-based fluorogenic and/or colorimetric probes for the selective detection of various analytes; (top) fluorescence turn “ON”; (middle) fluorescence turn “OFF”; (bottom) colorimetric sensor.

**Fig. 11:**
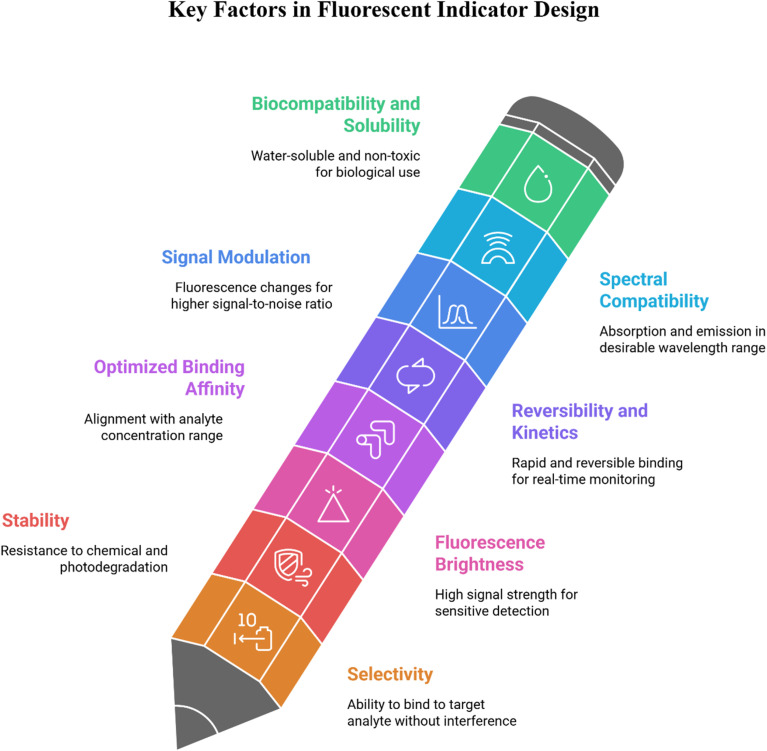
Design criteria for fluorescent sensors.

The design of BODIPY-based fluorescent sensors (Fig. 10) relies on the integration of a fluorophore with a recognition unit, enabling the modulation of optical output upon analyte binding.^85^ The functionality of these probes is predicated on various photophysical mechanisms that transduce a chemical interaction into a measurable signal (Fig. 11). Key mechanisms include photoinduced electron transfer (PET), Förster resonance energy transfer (FRET), intramolecular charge transfer (ICT), and excited-state intramolecular proton transfer (ESIPT), each providing distinct advantages for specific sensing applications.^86^

#### 2.2.1 Photoinduced electron transfer (PET)

**Fig. 12:**
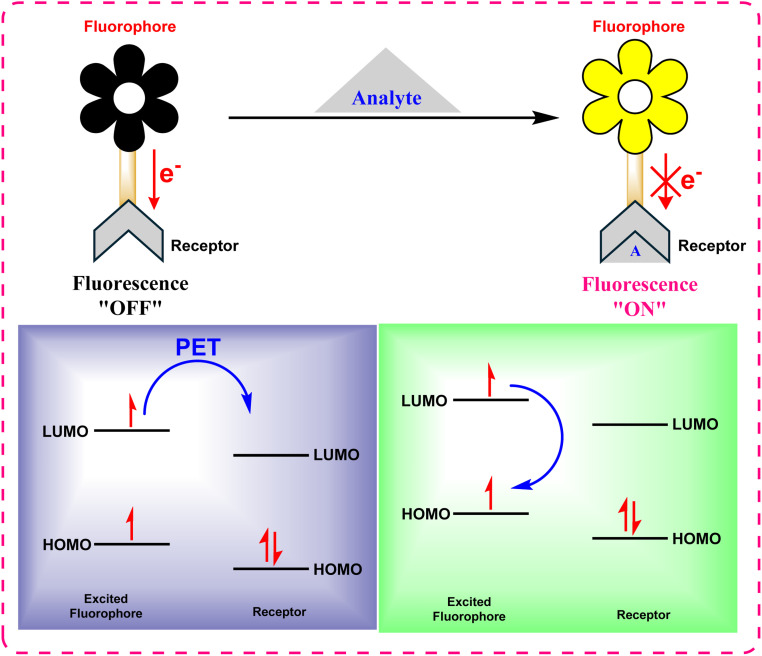
Mechanism of photoinduced electron transfer (PET).

In PET-based sensors, the fluorophore is electronically coupled to a recognition moiety. In the unbound state, photoexcitation is succeeded by transfer of electrons from the receptor to the fluorophore, a non-radiative process that quenches fluorescence (Fig. 12). The binding of a target analyte alters the redox potential of the receptor, thereby inhibiting this electron transfer pathway and restoring fluorescence emission. This mechanism effectively creates a “turn-on” or “turn-off” sensor highly sensitive to the presence of specific ions and biomolecules.^87^

#### 2.2.2 Intramolecular charge transfer (ICT)

**Fig. 13:**
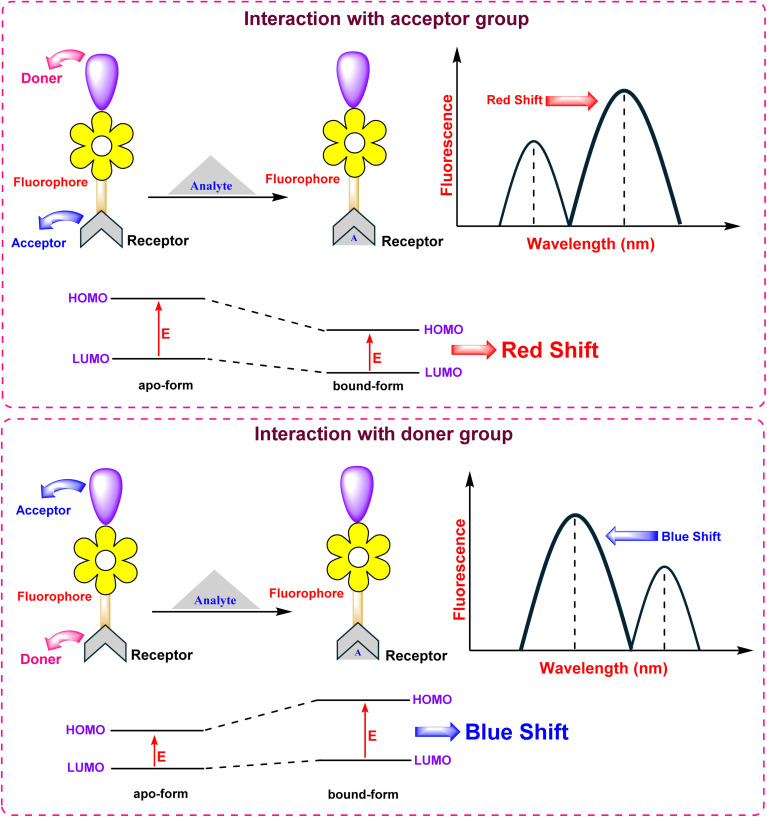
Mechanism of intramolecular charge transfer (ICT).

ICT-based sensors incorporate electron-donating (D) and electron-withdrawing (A) groups directly conjugated to the BODIPY core, creating a “push–pull” system (Fig. 13).^88^ Upon excitation, a significant charge redistribution occurs from the donor to the acceptor. Analyte interaction with either the D or A moiety modulates the efficiency of this charge transfer, resulting in distinct shifts in the absorption and emission spectra. This phenomenon allows for ratiometric sensing, where the ratio of fluorescence intensities at two different wavelengths provides a robust measurement that is independent of probe concentration and environmental fluctuations.

#### 2.2.3 Förster resonance energy transfer (FRET)

**Fig. 14:**
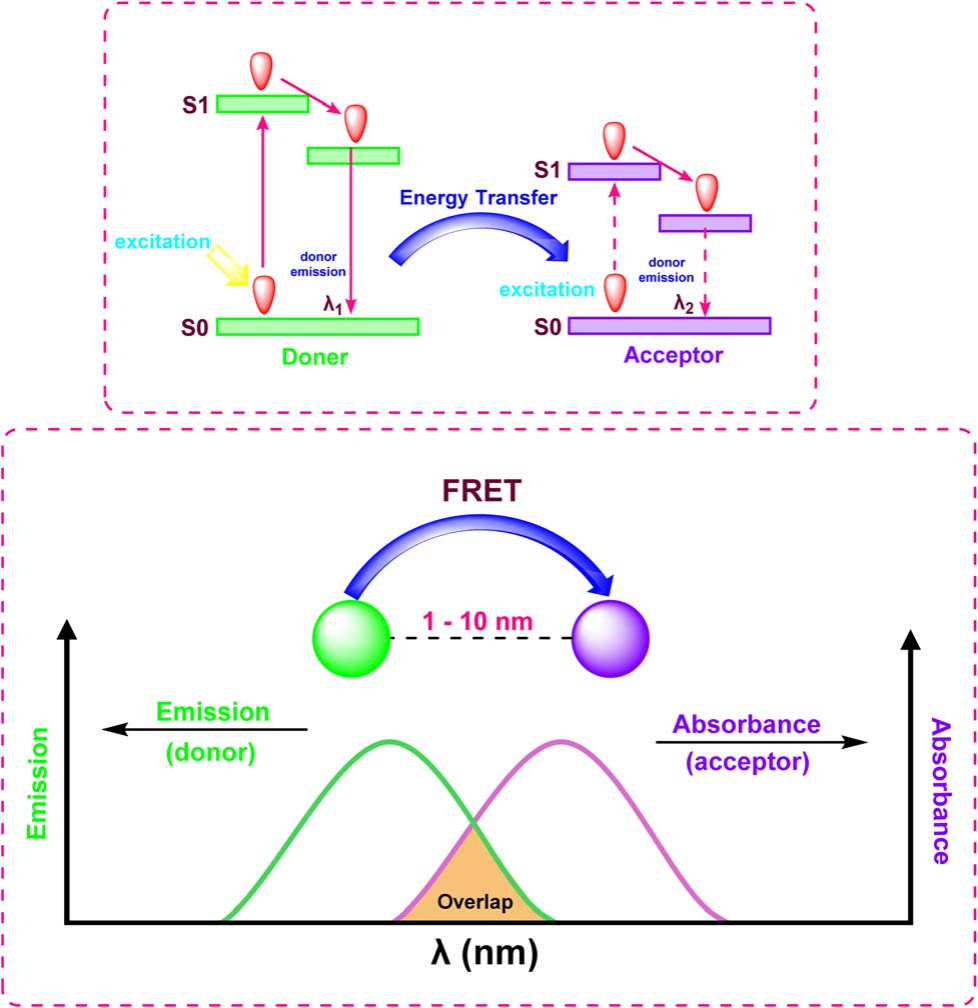
Mechanism of fluorescence resonance energy transfer (FRET).

FRET is a non-radiative process which involves transfer of energy from an excited donor fluorophore to an adjacent acceptor chromophore *via* dipole–dipole coupling (Fig. 14).^89^ The transfer efficiency decreases rapidly as the distance between the donor and acceptor increases, following an inverse sixth power law (*E*∝*r*^−6^), making it an exceptionally sensitive “spectroscopic ruler” for distances on the 1–10 nanometer scale. FRET is widely employed to monitor dynamic processes such as protein–protein interactions and changes in biomolecule conformation, often enabling ratiometric detection of analytes.

#### 2.2.4 Excited-state intramolecular proton transfer (ESIPT)

**Fig. 15:**
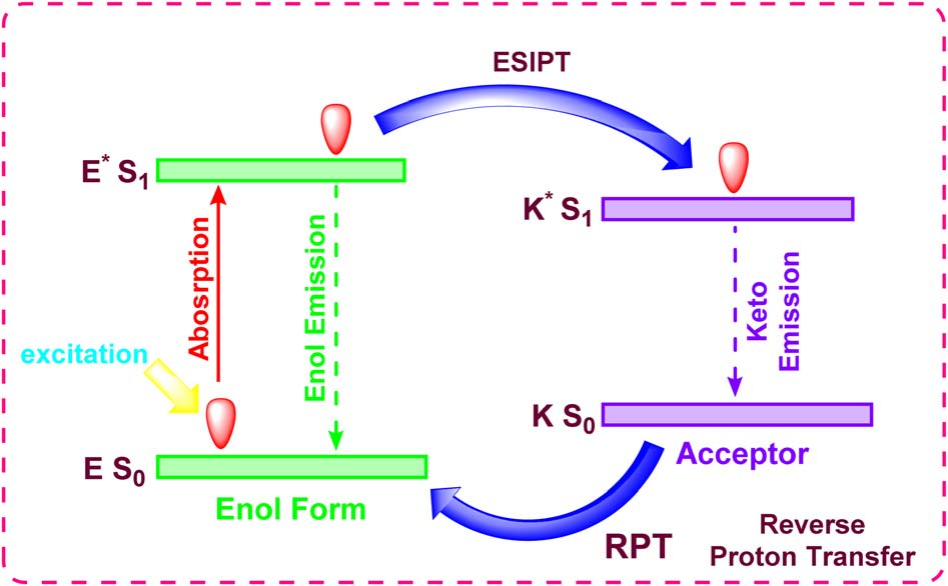
Mechanism of excited-state intramolecular proton transfer (ESIPT).

The ESIPT mechanism involves the rapid transfer of a proton within a molecule following photoexcitation, typically from a hydroxyl or amino group to an adjacent acceptor atom. This process generates a transient keto-tautomer that is electronically distinct from the initial enol form (Fig. 15).^90–92^ Because fluorescence emission occurs from this lower-energy keto state, ESIPT probes exhibit an unusually large separation between their absorption and emission wavelengths (a large Stokes shift). This characteristic is highly advantageous for creating sensitive fluorescent probes that minimize signal interference from self-absorption and scattered excitation light, making them well-suited for bioimaging and optoelectronic applications.

**Scheme 5:**
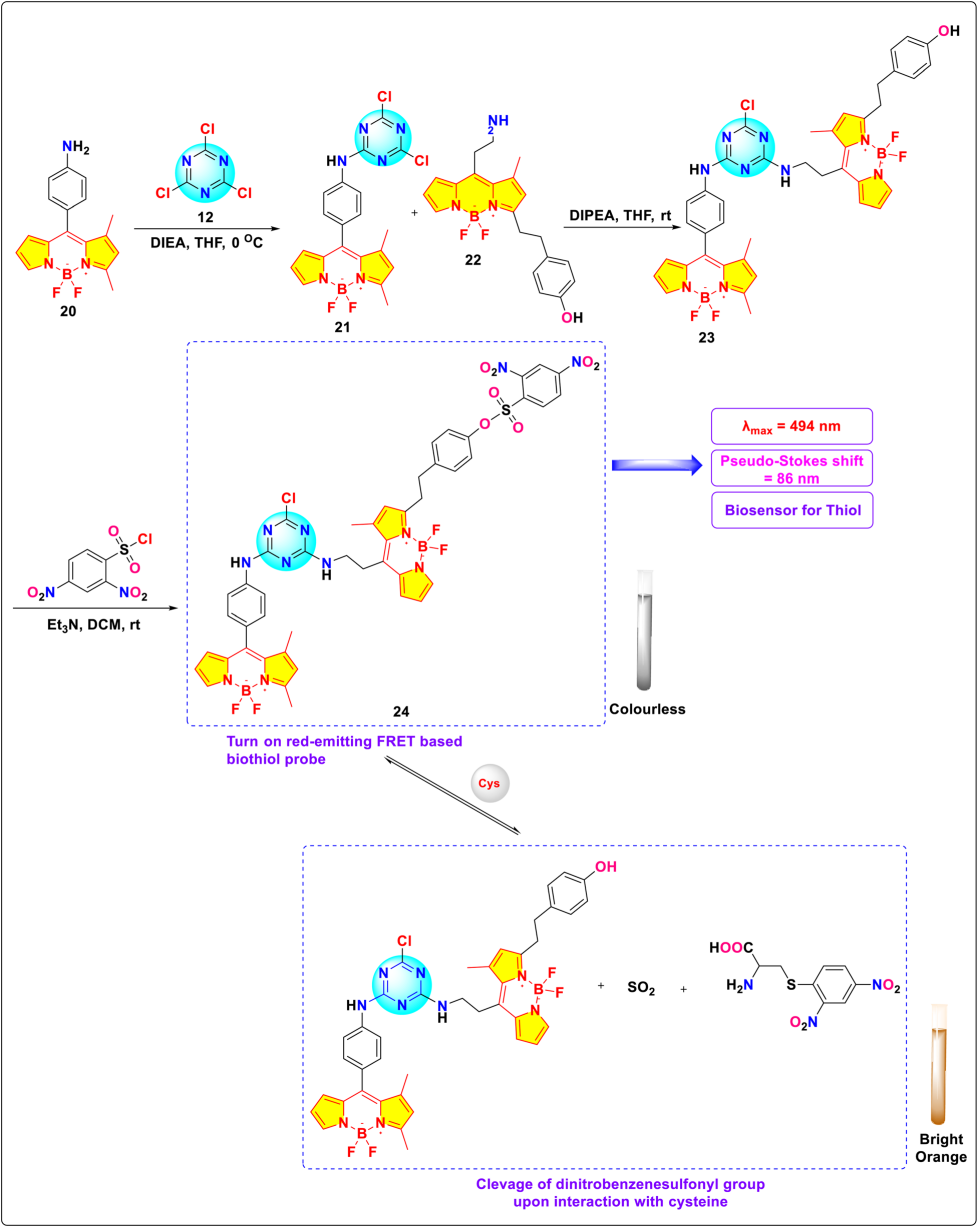
Synthesis and probable sensing mechanism of *s*-triazine-BODIPY conjugate 24.

**Fig. 16:**
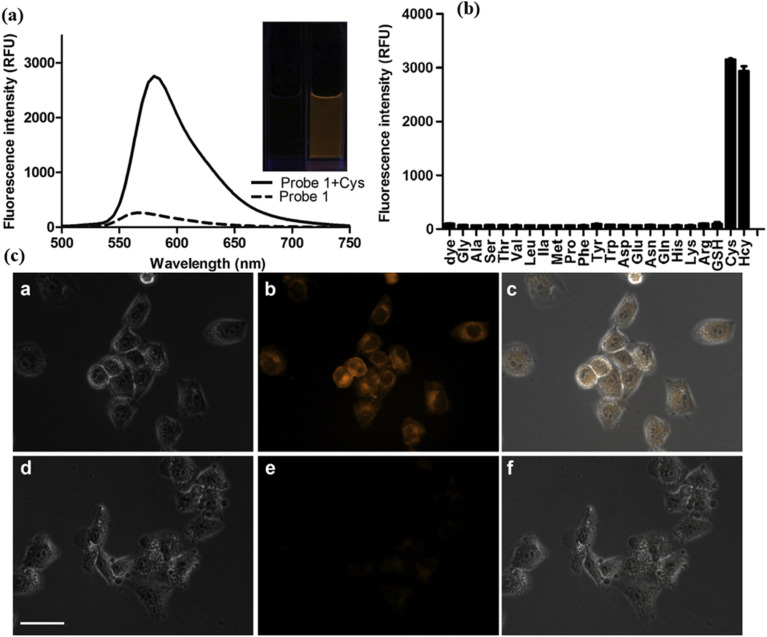
(a) Fluorescent emission spectra of probe 24; (b) fluorescence response of probe 24 to different analytes; (c) fluorescent microscopic images of HeLa cells pre-treated with *N*-methyl-maleimide and then incubated with probe 24. Reproduced with permission from ref. 85. Copyright 2014, Elsevier.

Dongdong and colleagues synthesized red-emitting *s*-triazine-BODIPY conjugate 24 designed for the detection of cysteine (Cys) and homocysteine (Hcy) *via* a fluorescence resonance energy transfer (FRET)-based “turn-on’’ mechanism (Scheme 5).^93^ The conjugate's sensing mechanism relies on the cleavage of the 2,4-dinitrobenzene sulfonyl (DNBS) protecting group from the fluorophore upon interaction with these thiols, resulting in enhanced fluorescence emission. Specifically, in the presence of Cys, a substantial 12-fold increase in fluorescence intensity at 580 nm occurs upon cleavage (Fig. 16). This transformation is accompanied by a discernible shift in emission from a non-fluorescent state to a bright orange emission. Furthermore, the bioimaging study on HeLa cells pretreated with *N*-ethylmaleimide (NEM) displayed that conjugate 24 can readily permeate cellular membranes to identify thiol species in living cells.

**Scheme 6:**
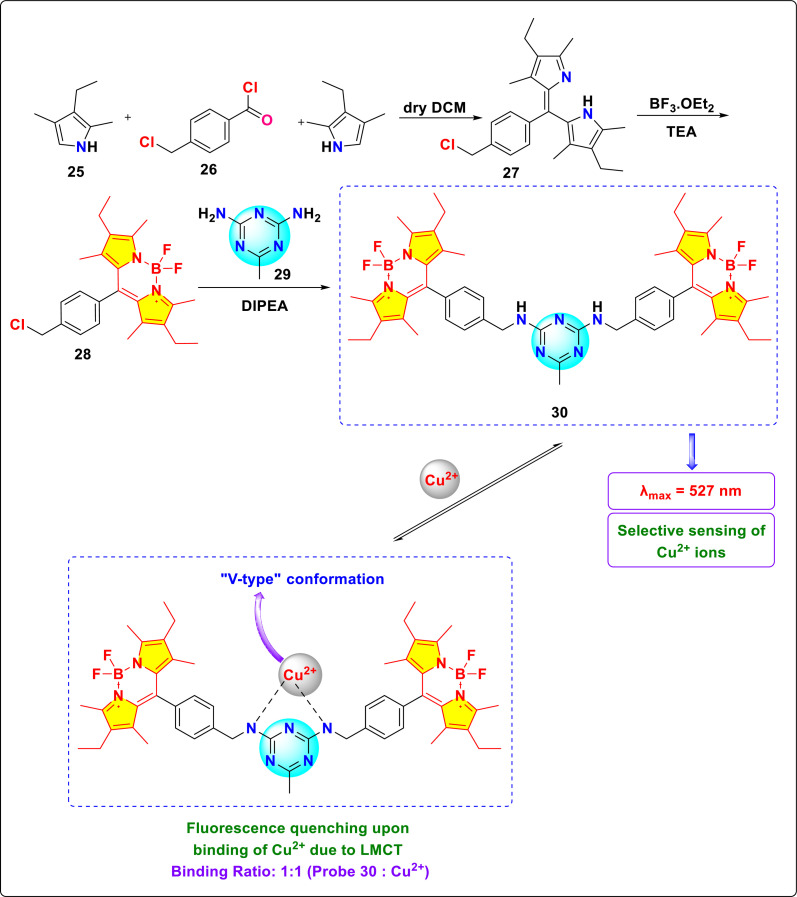
Synthesis and plausible sensing mechanism of *s*-triazine-BODIPY conjugate 30.

**Fig. 17:**
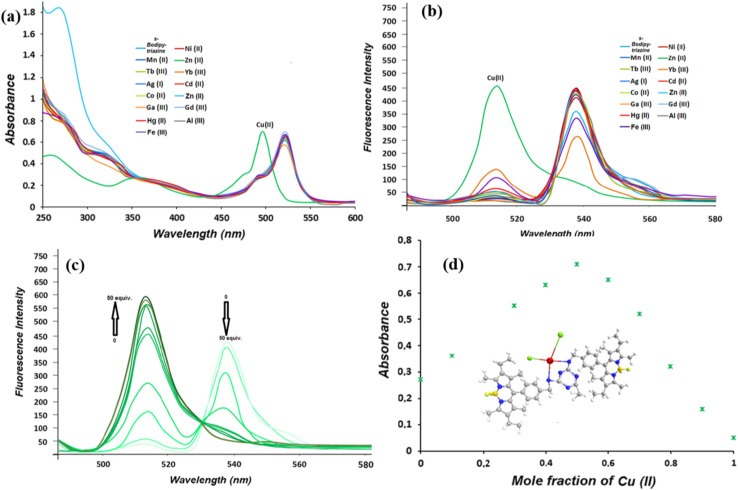
(a) Absorbance spectra of conjugate 30 with various metal ions; (b) fluorescence spectra of conjugate 30 with various metal ions; (c) Cu(ii) titration of conjugate 30 at different concentrations in DMF/H_2_O; (d) Job's Plot of conjugate 30/Cu(ii) reproduced with permission from ref. 86. Copyright 2014, Elsevier.

Kursunlu and colleagues developed a *s*-triazine-BODIPY conjugate 30, which has a high affinity towards Cu^2+^ ions in DMF : H_2_O (1 : 10, v/v) (Scheme 6).^94^ Upon the interaction of Cu^2+^ with the nitrogen atom of *s*-triazine, the resulting complex demonstrates a notable reduction in intensity at *λ*_max_ = 538 nm, along with a shift toward shorter wavelengths at *λ*_max_ = 513 nm in the DMF : H_2_O solvent system (Fig. 17). The decrease in emission intensity at a wavelength of 538 nm is attributed to the formation of a rigid framework upon binding with Cu^2+^, resulting in an enhanced fluorescence effect known as CHEF (Chelation enhanced fluorescence). This phenomenon can also be explained by the prevention of PET. The quenching constant, *K*_d_ = 7.5 × 10^−6^ was derived using the Stern–Volmer equation.

**Scheme 7:**
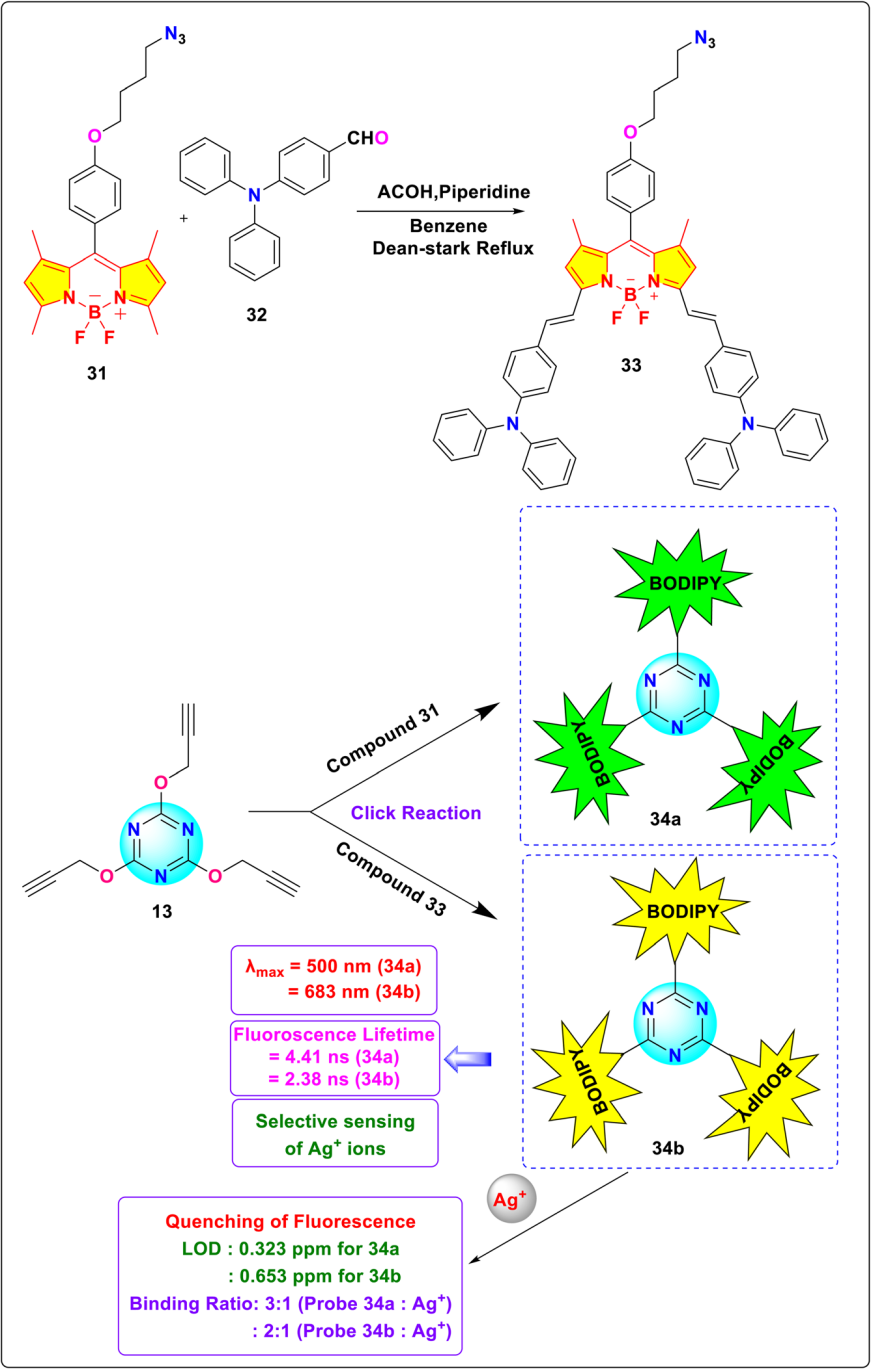
Synthetic route for *s*-triazine-BODIPY conjugates 34a and 34b.

**Fig. 18:**
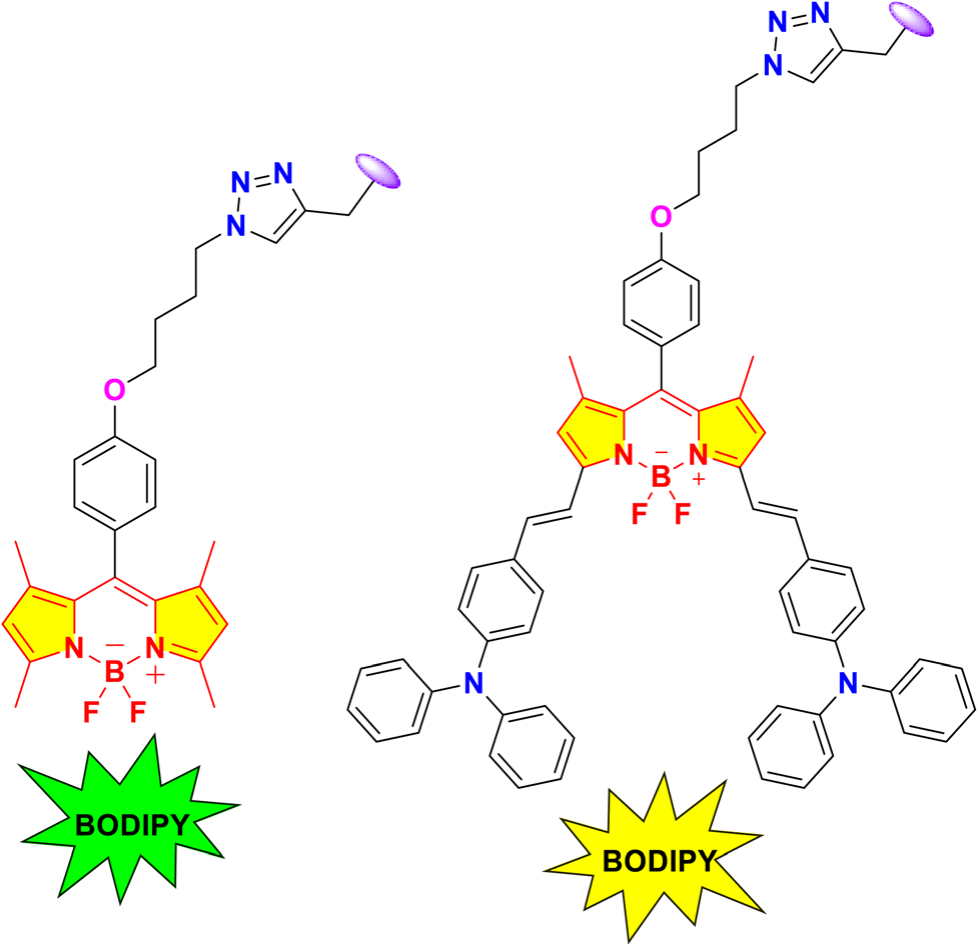
Structure of different BODIPY utilized in conjugates 34a and 34b.

E. Şenkuytu and group synthesized novel *s*-triazine-BODIPY conjugates 34a and 34b for the detection of silver (Ag^+^) ions in a THF: water medium (Scheme 7).^95^ They utilized the bright fluorescence of BODIPY compounds and the modulating properties of triazine derivatives to synthesize chemosensors for silver ions (Fig. 18).

The sensing capabilities of conjugates 34a and 34b towards various cations such as Cs^+^, Li^+^, Mg^2+^, Na^+^, Ca^2+^, Ba^2+^, K^+^, Fe^2+^, Mn^2+^, Ni^2+^, Co^2+^, Fe^3+^, Cu^2+^, Ag^+^, Cd^2+^, Cr^3+^, Al^3+^, Zn^2+^ and Hg^+^ were studied. The absorption maxima of the conjugates were observed at *λ*_max_ = 500 nm (34a) and 683 nm (34b). The emission spectra were acquired at *λ*_max_ = 511 nm (34a) and 720 nm (34b). The introduction of silver cations caused a substantial reduction in the fluorescence emission of probes 34a and 34b at a wavelength of 511 nm. Simultaneously, the other metal ions had a negligible effect. The triazine-BODIPY conjugates 34a and 34b exhibited a binding ratio of 3 : 1 (34a/Ag^+^) and 2 : 1 (34b/Ag^+^), respectively, as determined by Jobs' plot graph in a THF : water solution. The detection limit of the conjugates were measured to be 0.323 ppm (34a) and 0.653 ppm (34a). The Benesi–Hildebrand equation yielded association constants of 3.53 × 10^7^ M^−1^ (34a) and 8.16 × 10^6^ M^−1^ (34b) for the silver complexes, respectively.

### 2.3 Photophysical applications of *s*-triazine-BODIPY conjugates

Beyond the application of *s*-triazine-BODIPY conjugates in biological imaging, therapy, and chemical sensing, the unique photophysical characteristics lend themselves to a diverse range of applications in materials science and photochemistry. These applications utilize characteristics like robust visible light absorption, customizable fluorescence emission, effective energy transfer, responsiveness to environmental variations such as viscosity, and the capacity to produce reactive oxygen species. Researchers have investigated these conjugates in fields such as artificial photosynthesis, dye-sensitized and organic solar cells, molecular rotors for measuring viscosity, singlet oxygen production for photochemical applications, bioconjugation platforms, and liquid crystals.

**Scheme 8:**
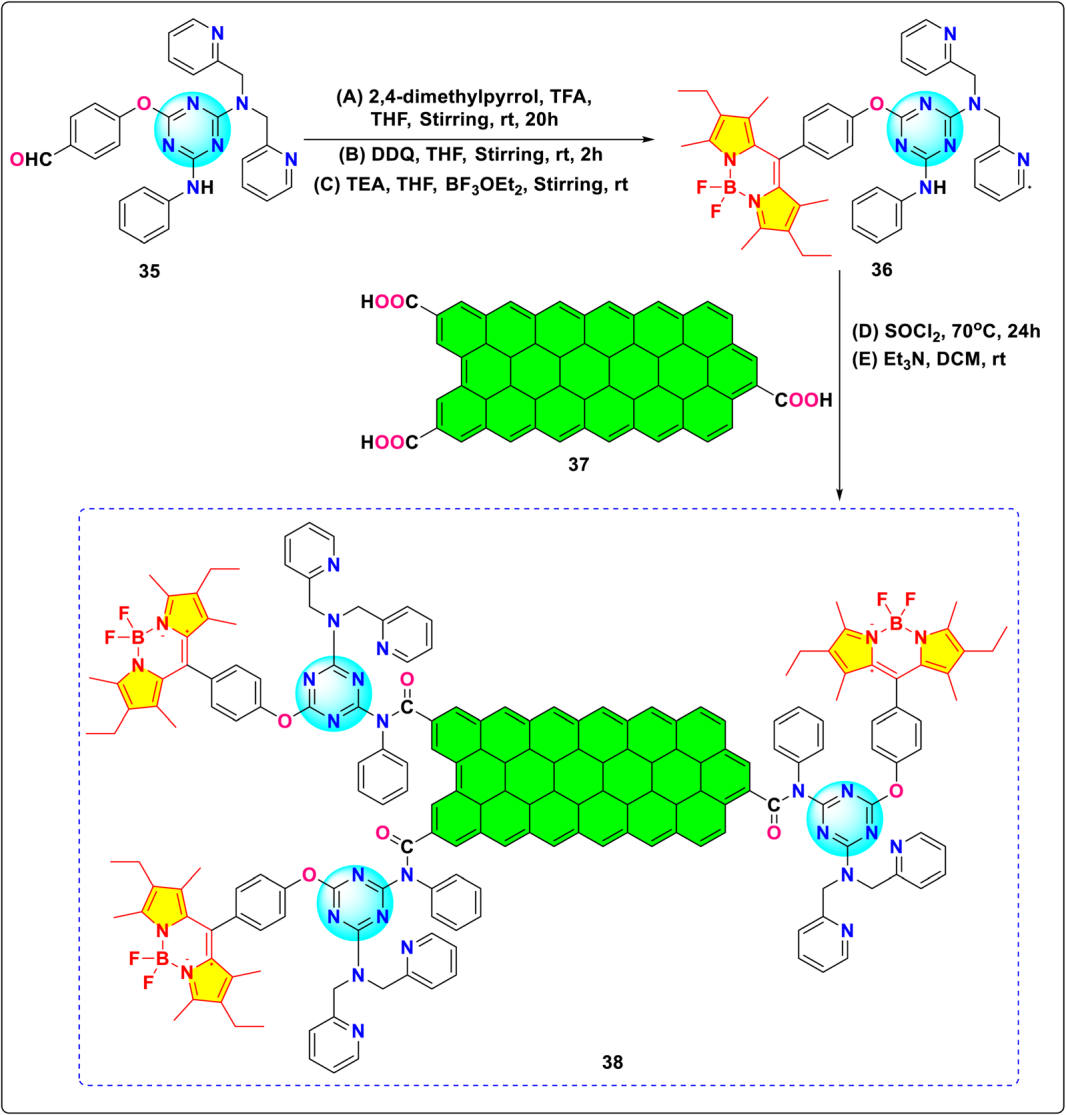
Synthetic route for *s*-triazine-BODIPY conjugate 38 for application in artificial photosynthetic system.

Baeg and colleagues demonstrated the application of functionalized graphene materials containing *s*-triazine-BODIPY conjugate 38 in artificial photosynthesis systems for coupled NADH regeneration and enzymatic carbon dioxide reduction (Scheme 8).^96^ The conjugate acts as a photocatalyst for NADH photoregeneration and CO_2_ fixation catalyzed by formate dehydrogenase (FDH) under visible light. The performance of the conjugate is reported to be better than the free multi-anthraquinonesubstituted porphyrin (MAQSP) system, showing 1.91 times the NADH production yield and generating 2.38 times more formic acid. The improved efficiency in these graphene-based systems is attributed to the graphene component's ability to promote efficient photoinduced charge separation and facilitate electron transfer within the integrated system.

**Scheme 9:**
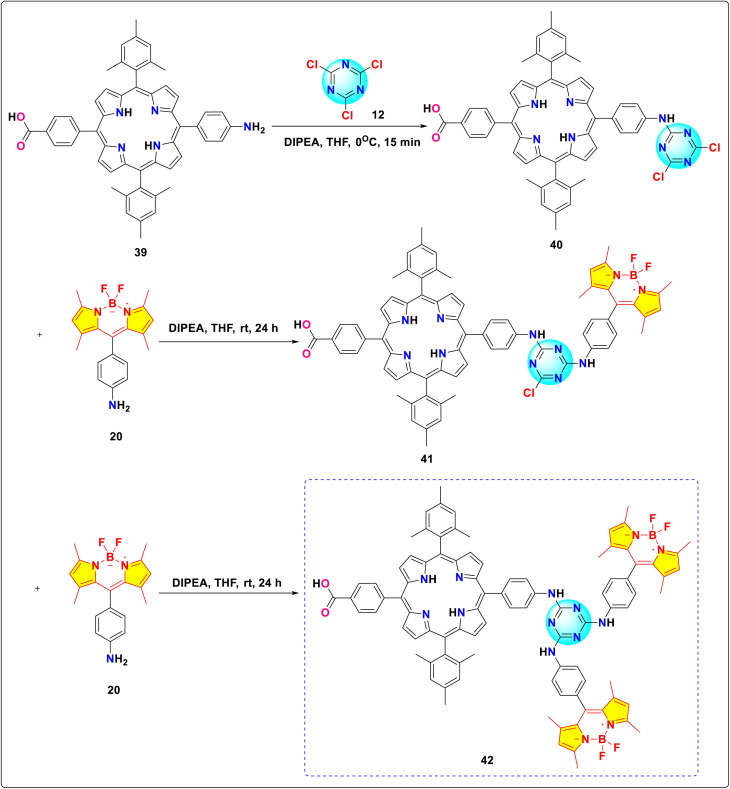
Synthetic route for *s*-triazine-BODIPY conjugate 42 for application in dye-sensitized solar cell.

**Fig. 19:**
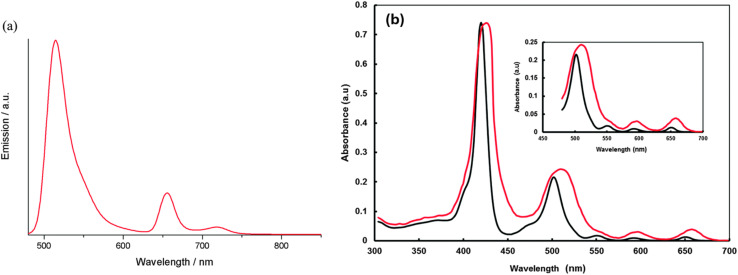
(a) Emission spectra of conjugate 42; (b) normalized absorption spectra of conjugate 42 in THF solution displayed in black line and adsorbed onto TiO_2_ film displayed in red line. Reproduced with permission from ref. 89. Copyright 2015, Royal Society of Chemistry.

Galateia *et.al.* synthesized *s*-triazine-BODIPY conjugate 42, containing two BODIPY moieties and one porphyrin moiety for dye-sensitized solar cell applications (Scheme 9).^97^ The conjugate exhibited significant absorption at 422 nm, 502 nm, 552 nm, 596 nm, and 602 nm (Fig. 19). The initial four peaks were designated to the porphyrin moiety, while the final peak was ascribed to the π to π* orbital transition of the BODIPY moiety. They found that conjugate 42 had a power conversion efficiency (PCE) of 5.17%, which improved to 6.20% when a thin layer of reduced graphene oxide was incorporated between the conjugate molecular layer and TiO_2_. Using ([6,6]-phenyl C71 butyric acid methyl ester) (PC71BM) as an acceptor and conjugate 42 as a donor, Sharma *et al.* developed a donor–acceptor framework to enhance the efficiency of the light-absorbing process in organic solar cells (OSC).^98^ The OSC device, composed of a 1 : 1 weight ratio of conjugate 42 and PC71BM in THF solvent, achieved a power conversion efficiency (PCE) of 5.29% and a short circuit current (*J*_SC_) of 10.48 mA cm^−2^.

**Scheme 10:**
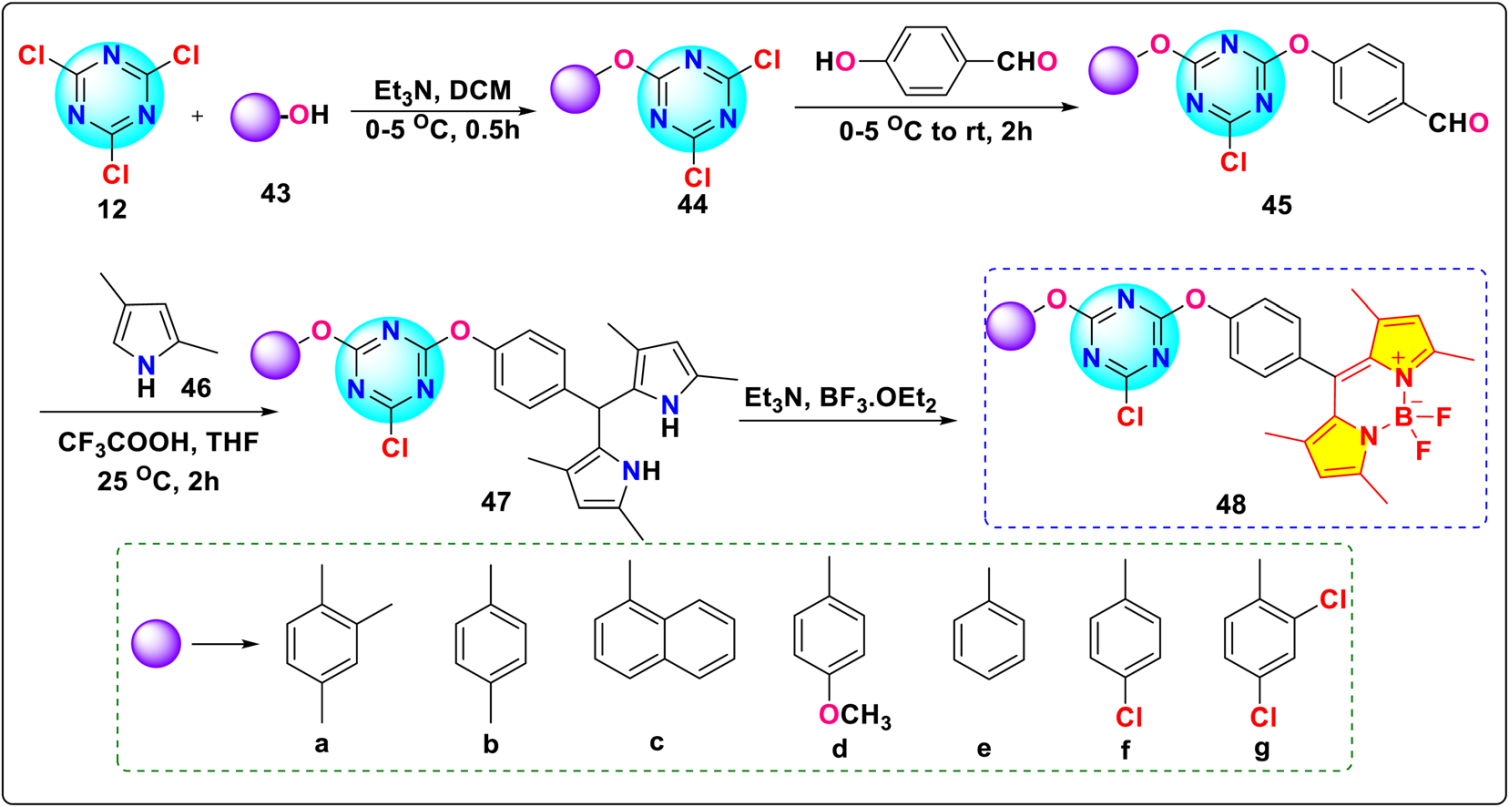
Synthetic route for *s*-triazine-BODIPY conjugates 48(a–g) for photophysical applications.

Jiahui *et al.* synthesized a novel *s*-triazine-BODIPY conjugates 48(a–g) incorporating seven mono-phenolic substituents (Scheme 10).^99^ The conjugates displayed absorption maxima between 499 and 501 nm in different solvents. Their Stokes shift was relatively small (Δ*ν* = 316 – 394 cm^−1^), with a high quantum yield between 0.46 and 0.65.

**Scheme 11:**
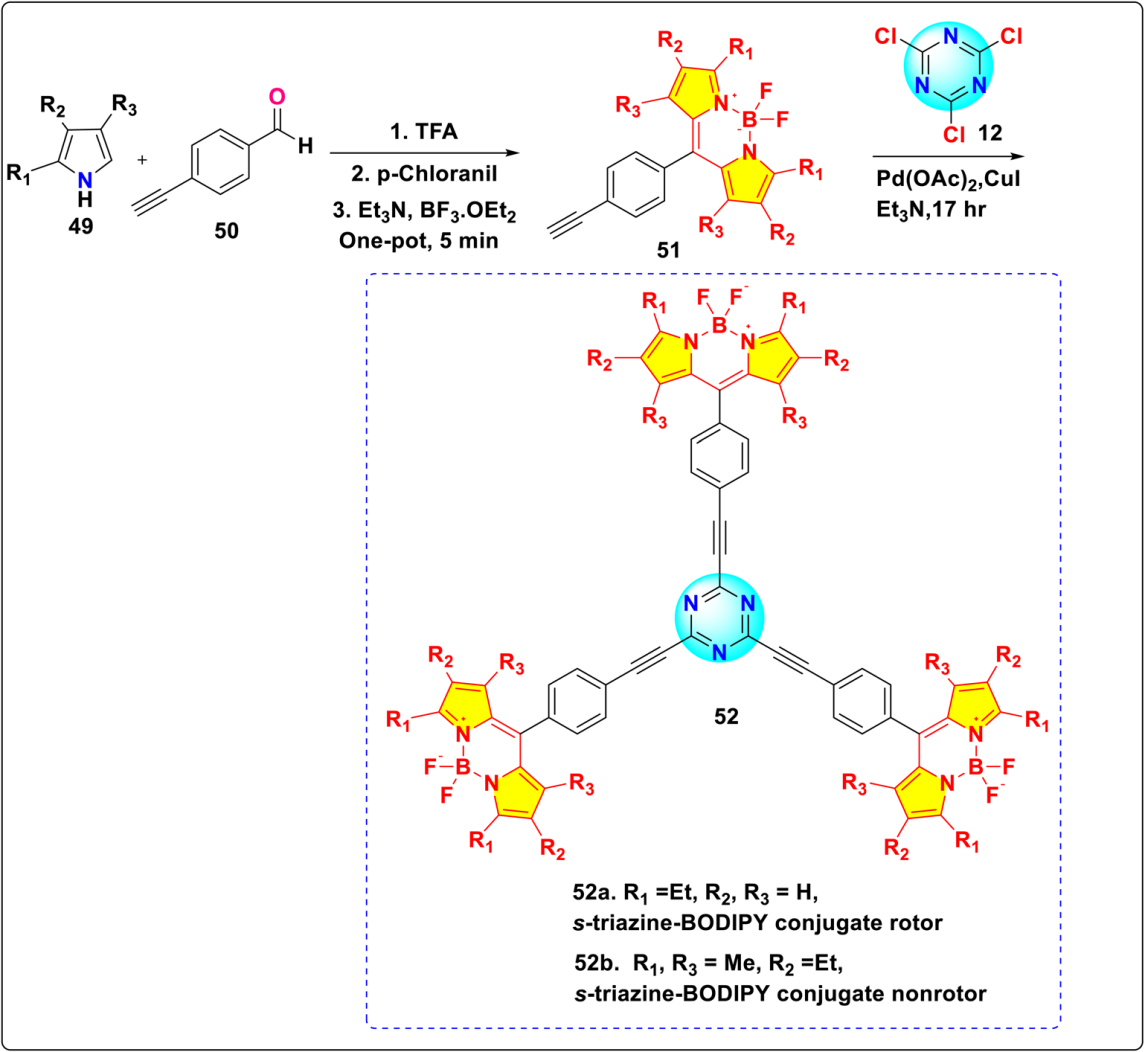
Synthetic route for *s*-triazine-BODIPY conjugates 52a and b for application as molecular viscometer.

**Fig. 20:**
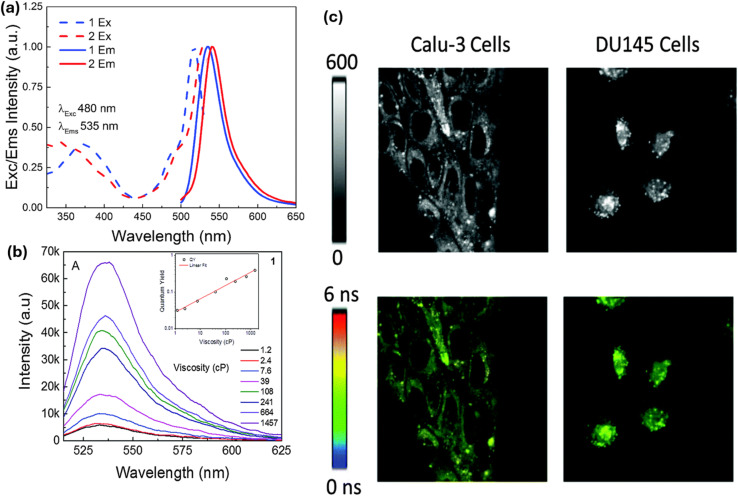
(a) Absorption (dotted line) and emission (solid line) spectra of conjugates 52a and b; (b) emission spectra of conjugates 52a in various viscosity mixtures of ethanol: glycerol; (c) FILM images of Calu3 and DU145 cells treated with 500 nM of conjugate 52a. Reproduced with permission from ref. 92. Copyright 2016, Royal Society of Chemistry.

Raut *et al.* reported the synthesis of *s*-triazine-BODIPY conjugates 52a and b, which can sense the viscosity change in molecular solvents, lipid vesicles, and several cancer cell lines (Scheme 11).^100^ Both the conjugates exhibited high molar absorption coefficients (around 200 000 M^−1^ cm^−1^), significantly higher than typical monomeric or dimeric rotors. Fluorescence intensity, quantum yield, and fluorescence lifetime of conjugate 52a increased significantly with increasing medium viscosity (Fig. 20). This results from the inhibition of non-radiative decay pathways associated with intramolecular rotation in viscous media environments. However, conjugate 52b showed minimal changes in emission intensity and lifetime with increasing medium viscosity due to hindrance in rotation. In cancer cells, conjugate 52a exhibited diffuse cytoplasmic accumulation and staining. *In vitro*, it interacted with larger proteins, such as BSA and HSA, leading to an increased fluorescence lifetime. However, it did not bind to smaller proteins such as lysozyme.

**Scheme 12:**
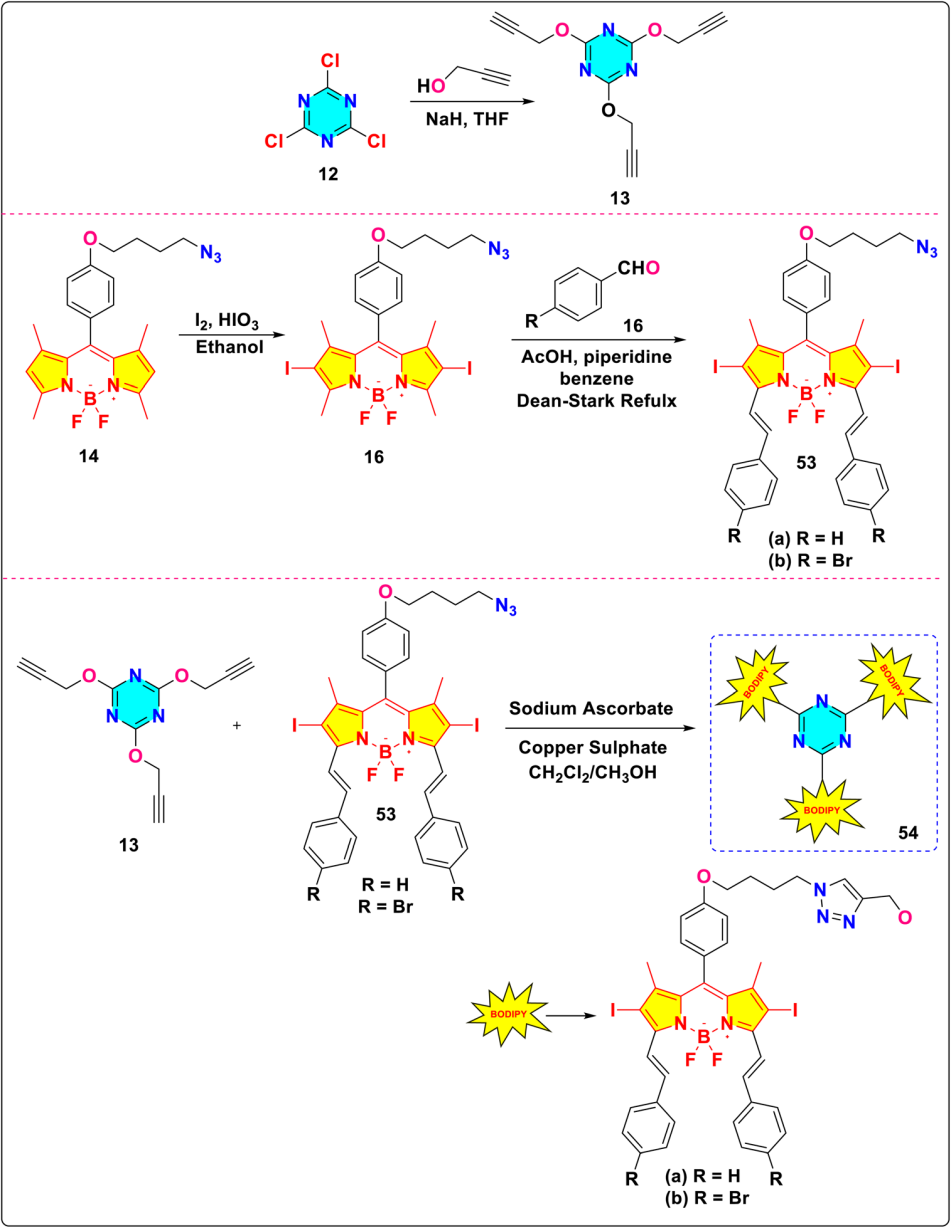
Synthetic route for *s*-triazine-BODIPY conjugates 54a and b as singlet oxygen generators.

Eçik *et al.* successfully designed and synthesized novel *s*-triazine-BODIPY conjugates 54a and b and evaluated their photochemical efficiency in generating singlet oxygen (Scheme 12).^101^ Both conjugates exhibited intense absorption maxima in the higher wavelength region of the spectrum (54a: 639 nm, 54b: 643 nm) with high molar extinction coefficients (54a: 8.3 × 10^4^ M^−1^ cm^−1^; 54b: 13.6 × 10^4^ M^−1^ cm^−1^). 54a and b displayed fluorescence quantum yields of 0.27 and 0.36 with lifetimes of 1.29 ns and 1.68 ns in CH_2_Cl_2_. Both conjugates demonstrated considerable efficiency as singlet oxygen generators, significantly surpassing the standard Methylene Blue. 54a and b displayed an exceptionally high efficiency of singlet oxygen production (*φ*Δ) of 0.82 and 0.75, respectively. This can be attributed to the heavy atom effect produced by iodine, which enhances intersystem crossing (ISC) from the singlet excited state to the triplet excited state. This phenomenon subsequently facilitates the transfer of energy to ground-state oxygen.

**Scheme 13:**
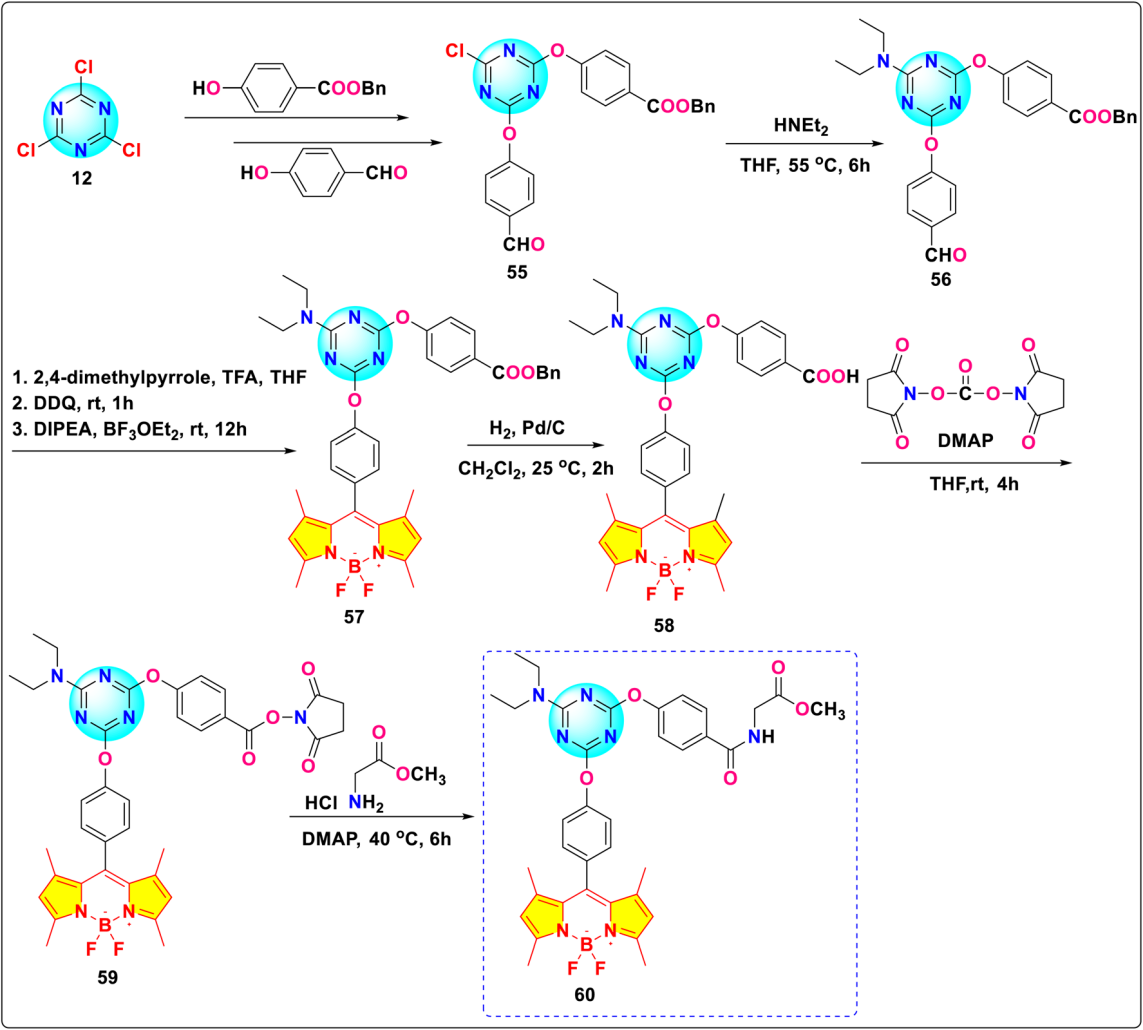
Synthetic route for *s*-triazine-BODIPY conjugate 60.

Shen and colleagues presented the successful synthesis of the *s*-triazine-BODIPY conjugate 59, containing a functionalized arm bearing an *N*-hydroxysuccinimide (NHS) ester (Scheme 13).^102^ They activated carboxylic acid compound 58 using *N*,*N*′-disuccinimidyl carbonate (DSC), producing the NHS ester dye (compound 59). This compound then easily underwent amidation with glycine methyl ester, resulting in conjugate 60. The simplicity of the amidation process demonstrates its effectiveness for bioconjugation chemistry, showcasing its potential for linking peptides, proteins, or other biomolecules. The absorption and emission spectra for compounds 58, 59, and conjugate 60 were measured in THF, revealing absorption and emission maxima near 500 nm and 511 nm, respectively.

**Scheme 14:**
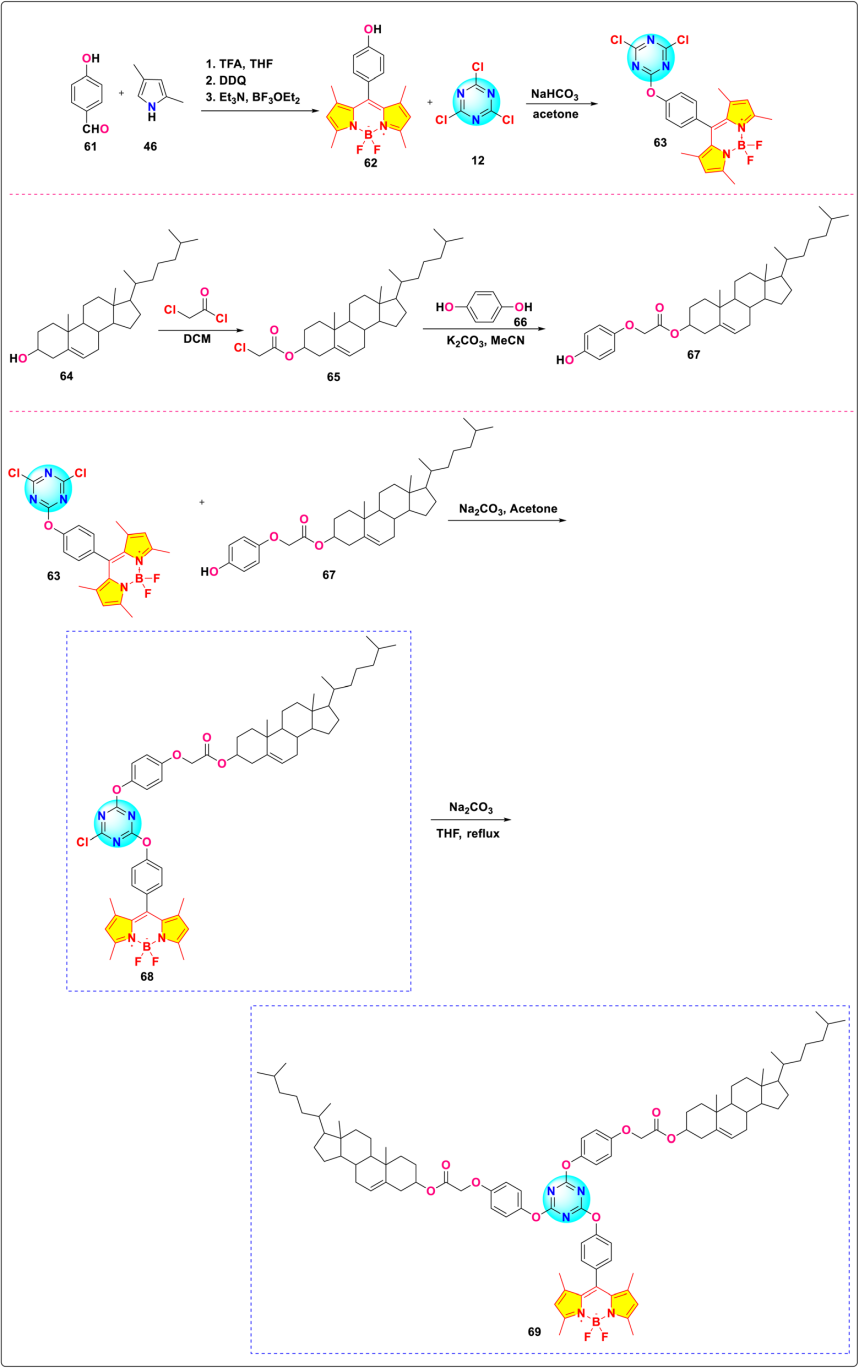
Synthetic route for *s*-triazine-BODIPY conjugates 68 and 69 for application as liquid crystal.

**Fig. 21:**
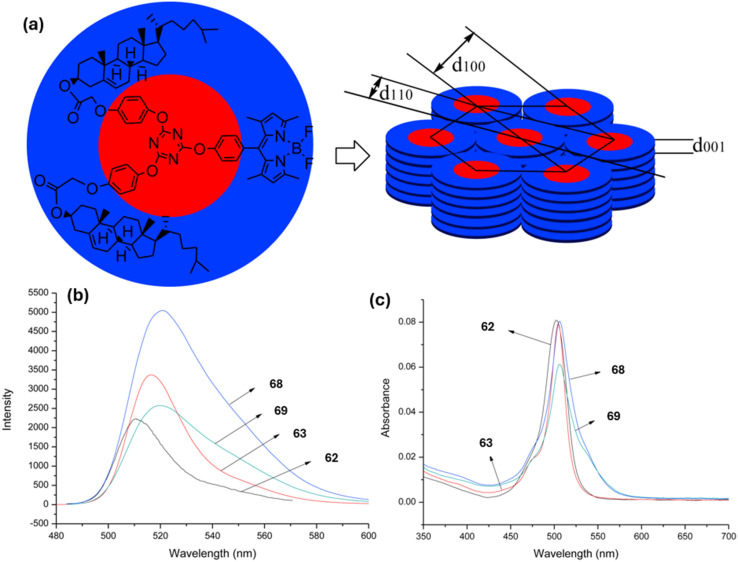
(a) Hexagonal columnar liquid crystal of conjugate 69; (b) absorption spectra of compounds 62, 63, and conjugates 68 and 69; (c) emission spectra of compounds 62, 63, and conjugates 68 and 69. Reproduced with permission from ref. 95. Copyright 2018, Elsevier.

Xiong and group reported the successful synthesis of two *s*-triazine-BODIPY conjugates 68 and 69, incorporating one and two cholesterol units, respectively (Scheme 14).^103^ Both conjugates showed strong absorption around 504–505 nm, with a quantum yield of 0.98 and 0.95 for 68 and 69, respectively (Fig. 21). The derivative with one cholesterol unit, 68, exhibited nematic liquid crystal behavior, whereas the derivative with two cholesterol units, 69, displayed a hexagonal columnar liquid crystal structure. The study presented the initial instances of cholesterol-BODIPY liquid crystals, where the addition of a cholesterol unit enhanced both the liquid crystalline properties and the overall improvement fluorescence.

**Scheme 15:**
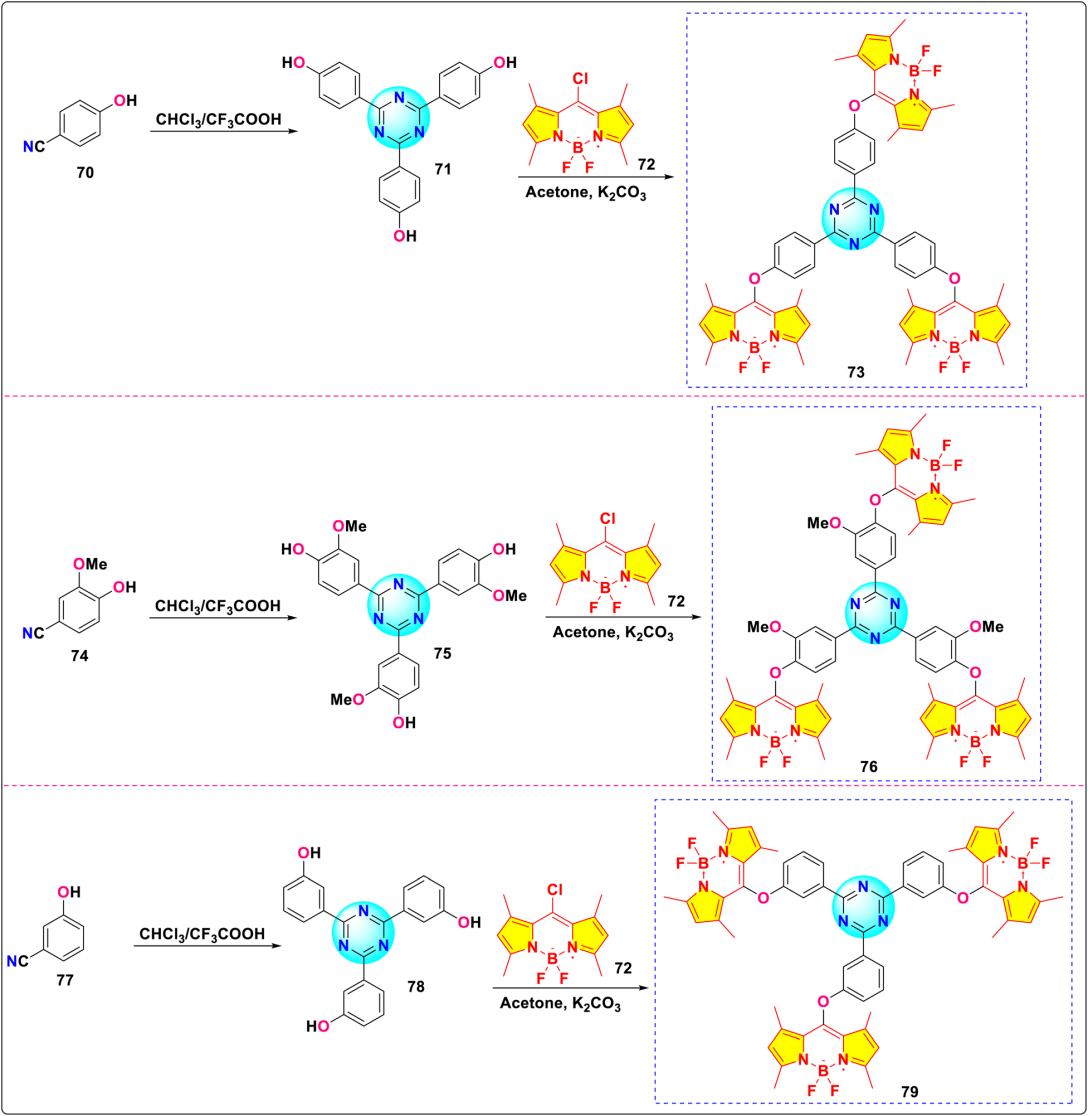
Synthetic route for *s*-triazine-BODIPY conjugates 73, 76, and 79 for photophysical study.

Maragani and co-workers synthesized a novel series of *s*-triazine-BODIPY conjugates 73, 76, and 79 to explore intramolecular interactions in both ground and excited states among the BODIPY units (Scheme 15).^104^ The normalized absorption and emission spectra of the aforementioned compounds were recorded using toluene. The conjugates exhibited absorption maxima approximately at 460 nm and emission maxima around 500 nm. The fluorescence lifetimes of conjugates 73, 76, and 79 were determined to be 5.56, 5.32, and 5.61 ns, respectively. In contrast to BODIPY derivatives featuring a meso-aryl substituent, the analogues functionalized with meso-*O*-aryl showed greater HOMO–LUMO gaps and Stokes shifts. This trend also affected singlet excited-state lifetimes, which were roughly doubled because of the direct addition of an electron-rich oxygen atom at the meso position. Analyses from spectroscopy, electrochemistry, and computational methods suggested minimal interactions in the ground state between the BODIPY entities within the trimers, which were symmetrically arranged around the triazine core. Among the three trimers investigated, only conjugate 76 displayed a weak degree of excitation transfer. Meso-*O*-aryl functionalized BODIPYs, with enhanced fluorescence, are promising for light-harvesting supramolecular oligomers and applications like sensing and imaging.

**Scheme 16:**
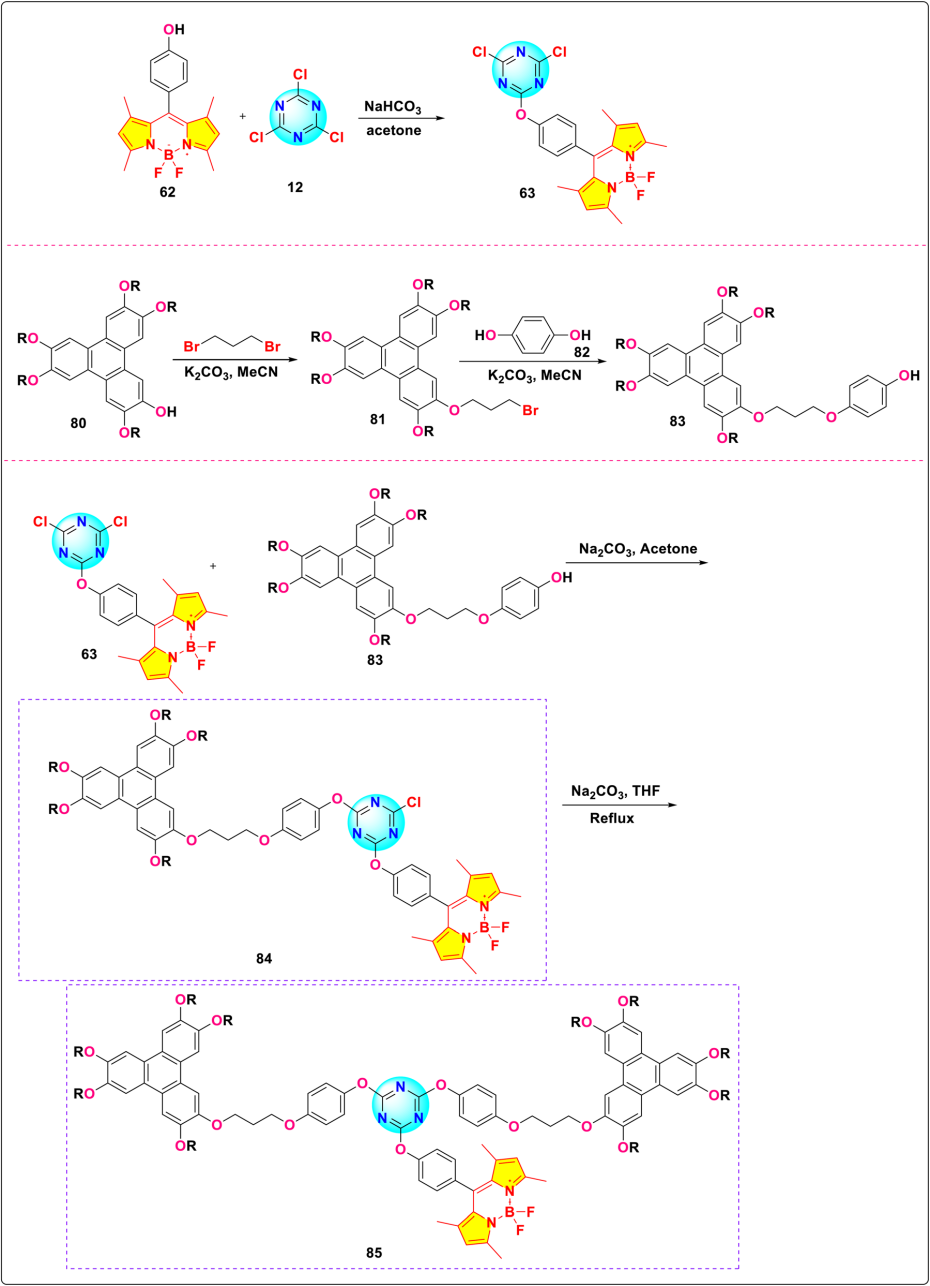
Synthetic route for *s*-triazine-BODIPY conjugates 84 and 85 for application as liquid crystal.

**Fig. 22:**
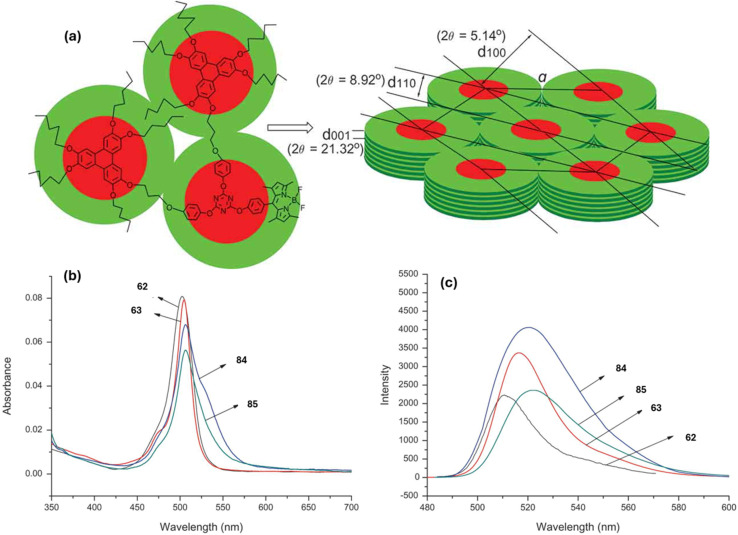
(a) Hexagonal columnar liquid crystal of conjugate 85; (b) absorption spectra of compounds 62, 63, and conjugates 84 and 85; (c) emission spectra of compounds 62, 63, and conjugates 84 and 85. Reproduced with permission from ref. 97. Copyright 2018, Taylor and Francis.

Feng *et al.* synthesized two *s*-triazine-BODIPY conjugates, 84 and 85, and investigated their mesomorphic properties (Scheme 16).^105^ Mesomorphic studies revealed that conjugate 84, incorporating a single triphenylene moiety, exhibited a nematic liquid crystal, whereas conjugate 85, bearing two triphenylene units, formed a hexagonal columnar liquid crystal. Both conjugates displayed absorption maxima (*λ*_max_) around 506 nm, along with a high quantum yield of 0.95 for conjugate 84 and 0.92 for conjugate 85 (Fig. 22). The conjugates also show a large Stokes shift (15 nm for 84, 17 nm for 85) attributed to enhanced Intramolecular Charge Transfer (ICT).

**Scheme 17:**
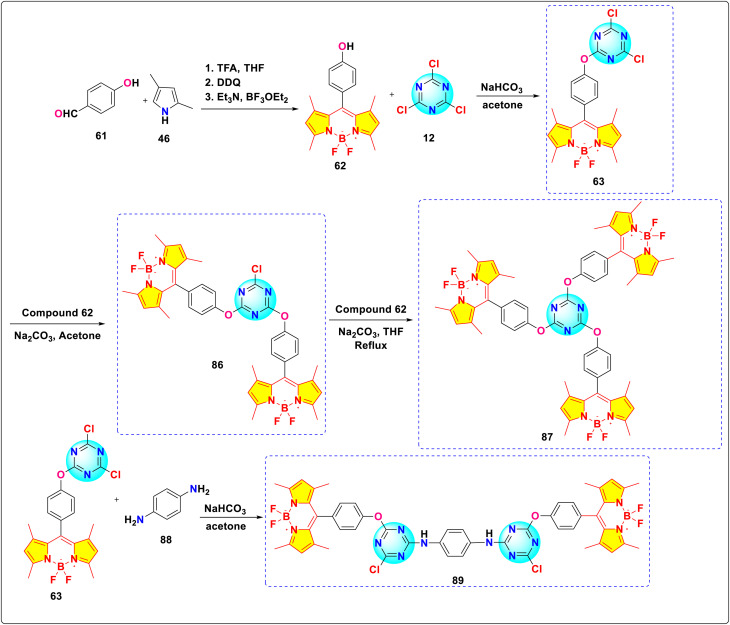
Synthetic route for *s*-triazine-BODIPY conjugates 63, 86, 87, and 89 for molecular viscometer application.

Zhou *et al.* synthesized a series of novel *s*-triazine-BODIPY conjugates 63, 86, 87, 89 and investigated the effect of increasing the number of BODIPY units on photophysical properties (Scheme 17).^106^ Among these derivatives, conjugate 86, featuring two BODIPY units, exhibited the most intense fluorescence with fluorescence quantum yields (*Φ*_f_) of 0.15 in methanol (MeOH) and 0.29 in *N*,*N*-dimethylformamide (DMF). This enhanced emission is attributed to the combined fluorescence of the two BODIPY units and minimized H-aggregation resulting from its reduced molecular symmetry. However, conjugate 89, featuring two BODIPY units, demonstrated significant H-aggregation quenching owing to the strong π–π stacking interactions stemming from the greater molecular symmetry of its expansive, rigid bridging structure in contrast to conjugate 86. The addition of a third BODIPY unit (conjugate 87) leads to a decrease in fluorescence intensity and quantum yield, due to aggregation-induced quenching (ACQ) or H-aggregation effects between the multiple BODIPY units. Based on the findings of the emission spectra of conjugate 86, viscosity measurements revealed a linear correlation between viscosity and fluorescence intensity/steady-state emission anisotropy, consistent with Förster–Hoffmann theory. This correlation indicates that conjugate 86 may serve as a useful probe for detecting viscosity.

**Fig. 23:**
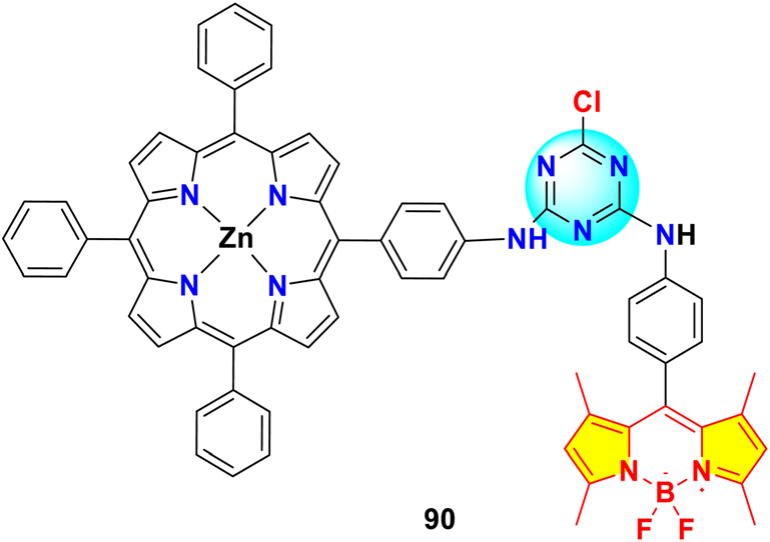
Structure of *s*-triazine-BODIPY conjugates 90 used in perovskite and organic solar cells.

Gkini *et al.* have enhanced the efficiency and reliability of perovskite solar cells (PSCs) and organic solar cells (OSCs) using *s*-triazine-BODIPY conjugate 90 as electron transfer materials (Fig. 23).^107^ The conjugate 90 exhibited absorption properties, with a Soret band at 422 nm linked to the S_0_ → S_2_ transition. Additionally, two Q bands appear at 558 and 598 nm, corresponding to the S_0_ → S_1_ transition. A separate absorption peak at 500 nm, attributed to the BODIPY moiety, also results from the S_0_ → S_1_ transition. The PSC device, fabricated using conjugate 90 as electron transfer mediator, showed a PCE of 17.34%, with a *J*_sc_ of 21.11 mA cm^−2^, *V*_oc_ of 1.11 V, and FF of 0.74.

**Scheme 18:**
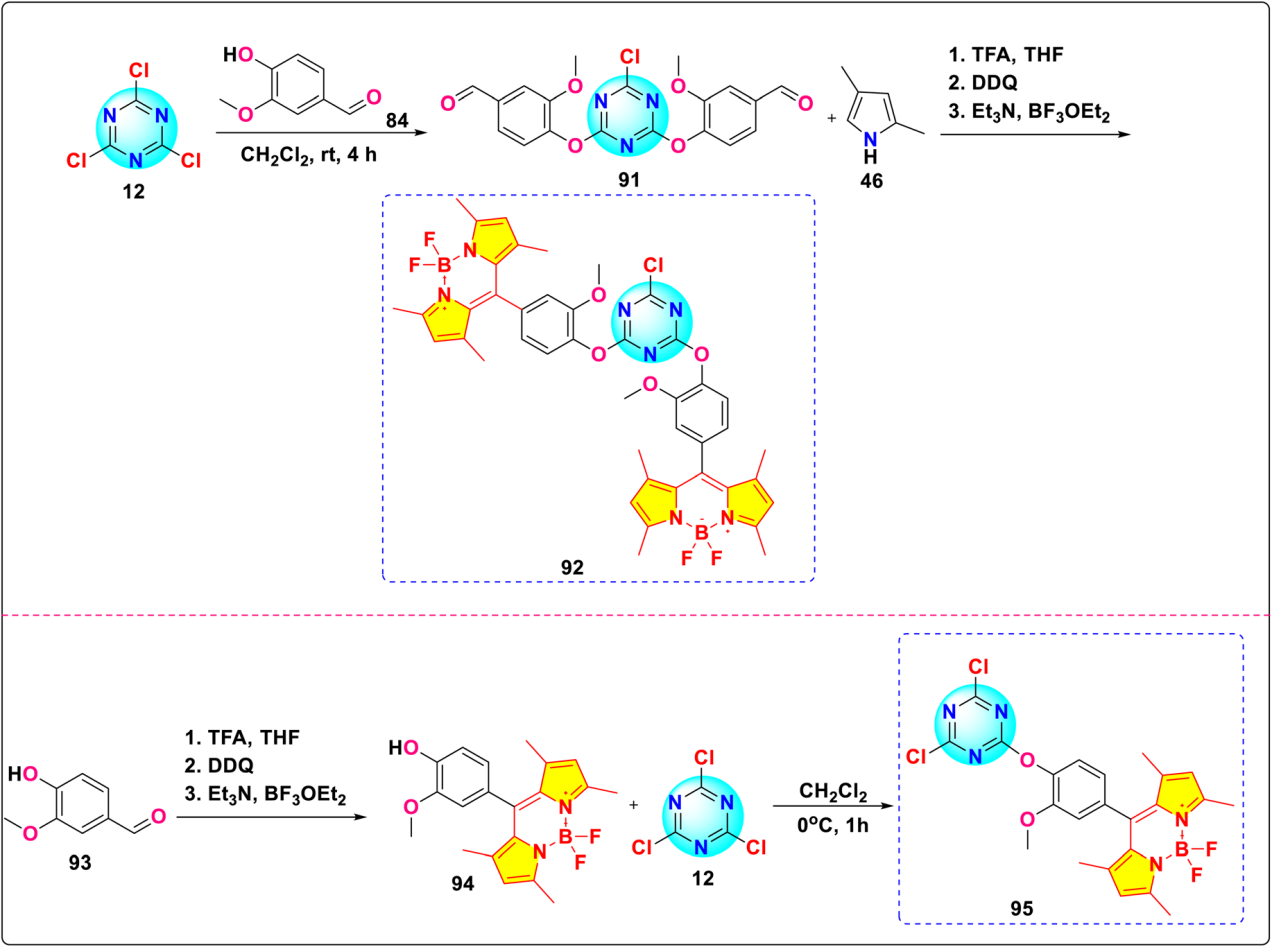
Synthetic route for *s*-triazine-BODIPY conjugates 92 and 95 for photophysical studies.

**Fig. 24:**
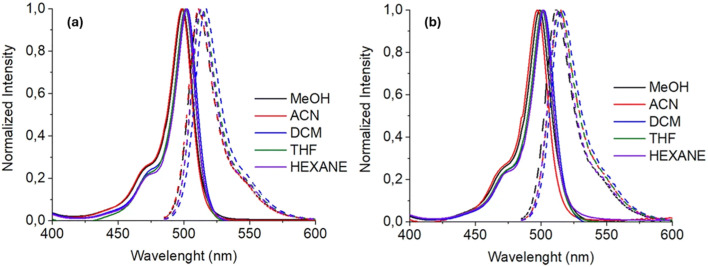
Absorption (solid line) and emission (dotted line) spectra of (a) conjugate 92 and (b) conjugate 95 in different solvents. Reproduced with permission from ref. 100. Copyright 2019, Springer Nature.

González and co-workers synthesized two novel *s*-triazine-BODIPY conjugates, 92 and 95 and studied their photophysical properties along with BODIPY compound 94 (Scheme 18).^108^ All the conjugates exhibited absorption maxima at around 500 nm, which is attributed to a strong S_0_ – S_1_ transition (Fig. 24). It was reported that the incorporation of the triazine core generally caused a hypsochromic (blue) shift in absorption (*λ*_abs_) and a bathochromic (red) shift in emission (*λ*_em_) due to an increase in the stability of the system and influences the π–π* transitions. Furthermore, the increase in the number of BODIPY units was associated with improved fluorescence, especially in polar solvents.

**Scheme 19:**
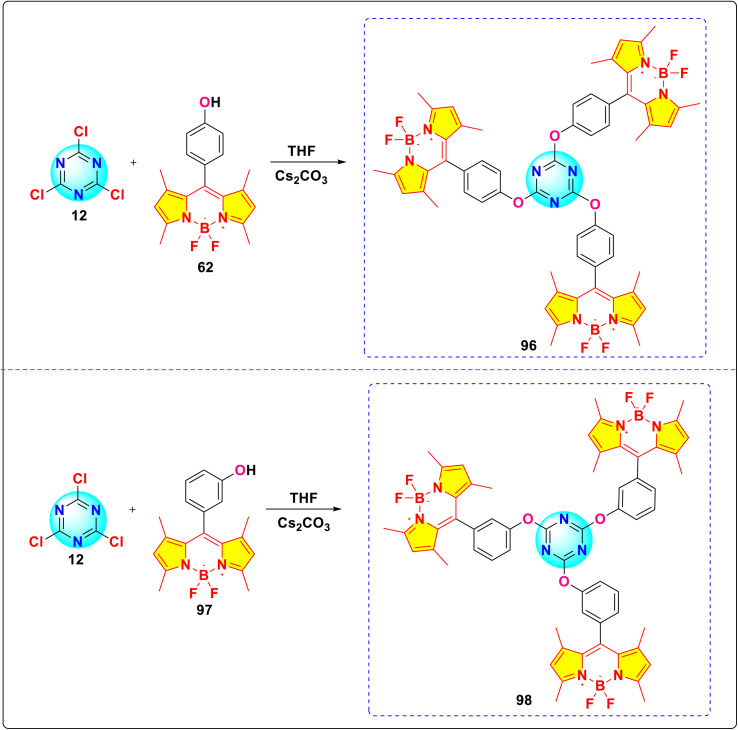
Synthetic route for *s*-triazine-BODIPY conjugates 96 and 98 for photodiode applications.

**Fig. 25:**
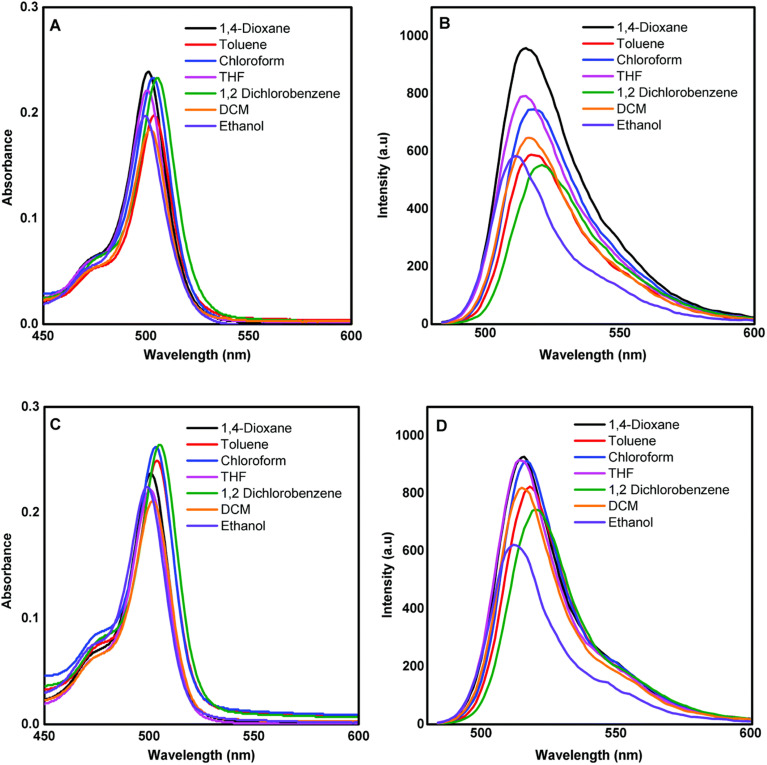
(a) Absorption spectra of conjugate 96 in different solvents; (b) emission spectra of conjugate 96 in different solvents; (c) absorption spectra of conjugate 98 in different solvents; (d) emission spectra of conjugate 98 in different solvents. Reproduced with permission from ref. 101. Copyright 2020, Royal Society of Chemistry.

Aksoy and colleagues reported the synthesis of novel *s*-triazine-BODIPY conjugates 96 and 98 and studied their photophysical and thermal properties (Scheme 19).^109^ Both tripods exhibited strong absorption around 500 nm and corresponding fluorescence emission, characteristic of BODIPY dyes (Fig. 25). The para-substituted conjugate 96 showed a slightly higher fluorescence quantum yield and longer lifetime than the meta-substituted conjugate 98 in DCM. The fabricated diodes (Al/conjugate/p-Si/Al) exhibited clear rectifying behavior in the dark. Under illumination, the current increased significantly, demonstrating a distinct photoresponse. The devices also displayed sensitivity to light intensity, and the position of the OH group (para *vs.* meta) did not drastically alter the overall photodiode characteristics.

**Scheme 20:**
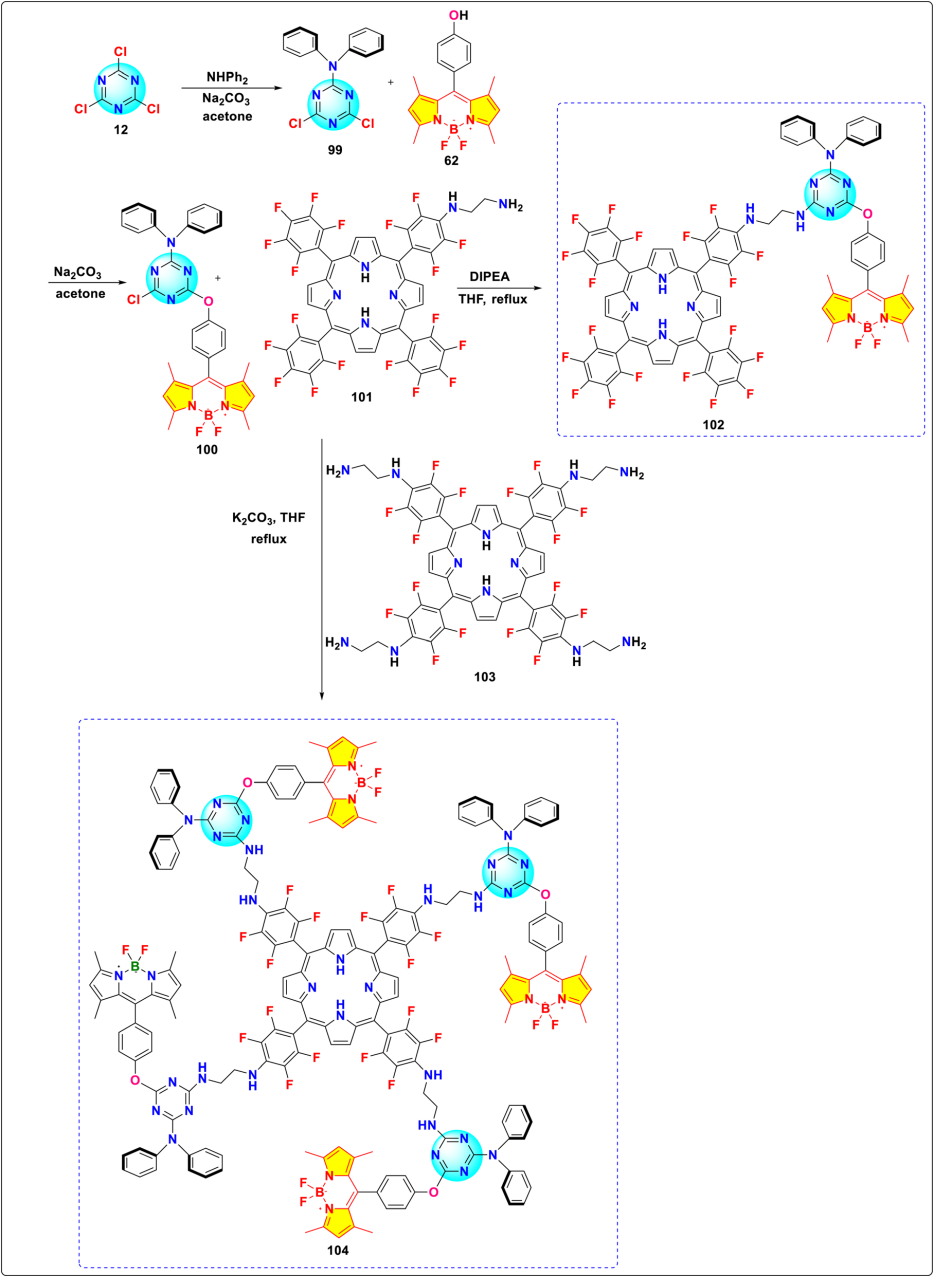
Synthetic route for *s*-triazine-BODIPY conjugates 102 and 104 for photophysical studies.

Souza and colleagues synthesized two novel *s*-triazine-BODIPY conjugates, 102 and 104, containing BODIPY (donor, D) and porphyrin (acceptor, A) units linked *via* a 1,3,5-triazine bridge (Scheme 20).^110^ Both conjugates showed distinct absorption peaks linked to the porphyrin Soret band (410–430 nm) and Q bands, along with a pronounced BODIPY absorption band (502 nm), showing an absence of conjugation between the porphyrin and BODIPY units. Steady-state and time-resolved fluorescence showed efficient FRET from BODIPY donors to porphyrin acceptors in both triads. Upon excitation with near-infrared (NIR) light at a wavelength of 930 nm, both the green emission characteristic of BODIPY, observed around 514 nm, and the red emission characteristic of porphyrin, observed within the range of 650 to 750 nm, were detected in conjugates 102 and 104. This demonstrates nonlinear up conversion where lower-energy NIR photons generate higher-energy visible emission.

**Scheme 21:**
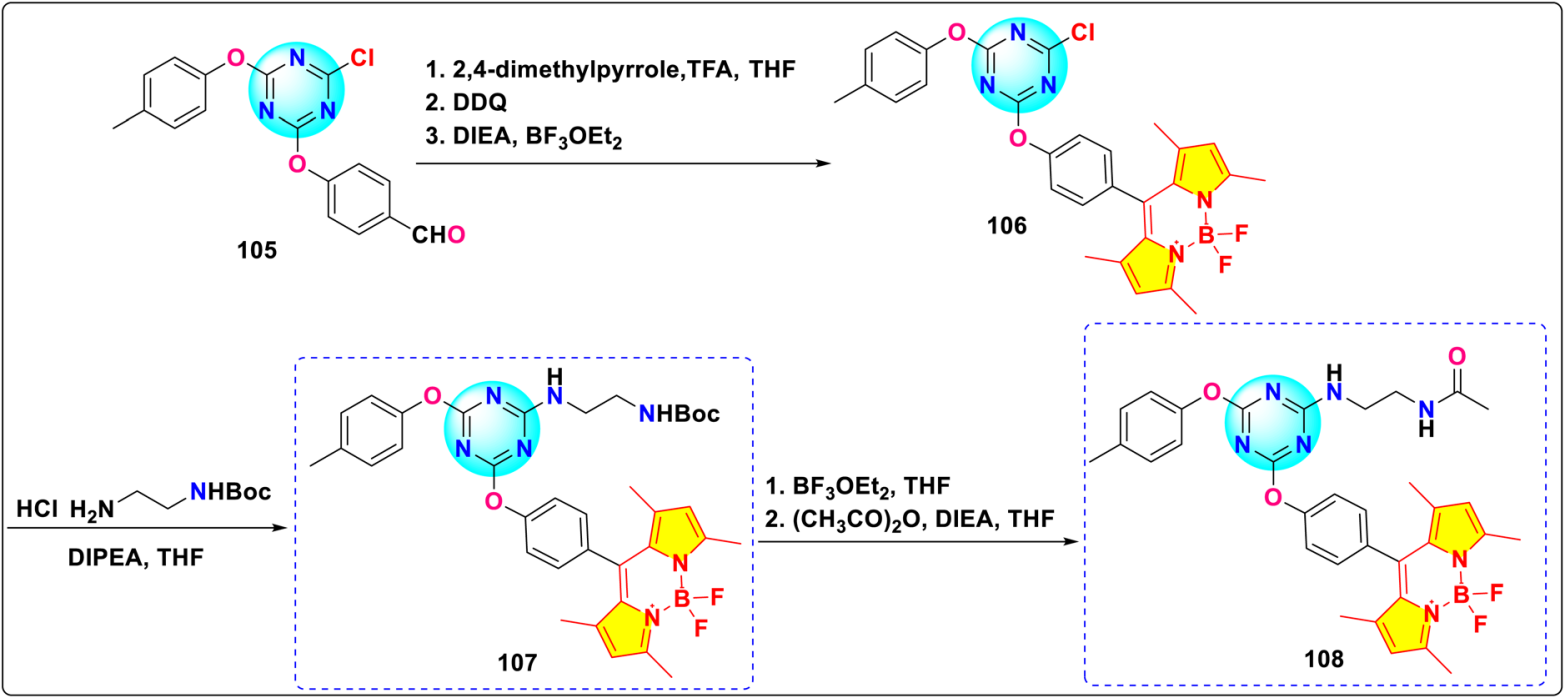
Synthetic route for *s*-triazine-BODIPY conjugates 107 and 108 for photophysical studies.

**Fig. 26:**
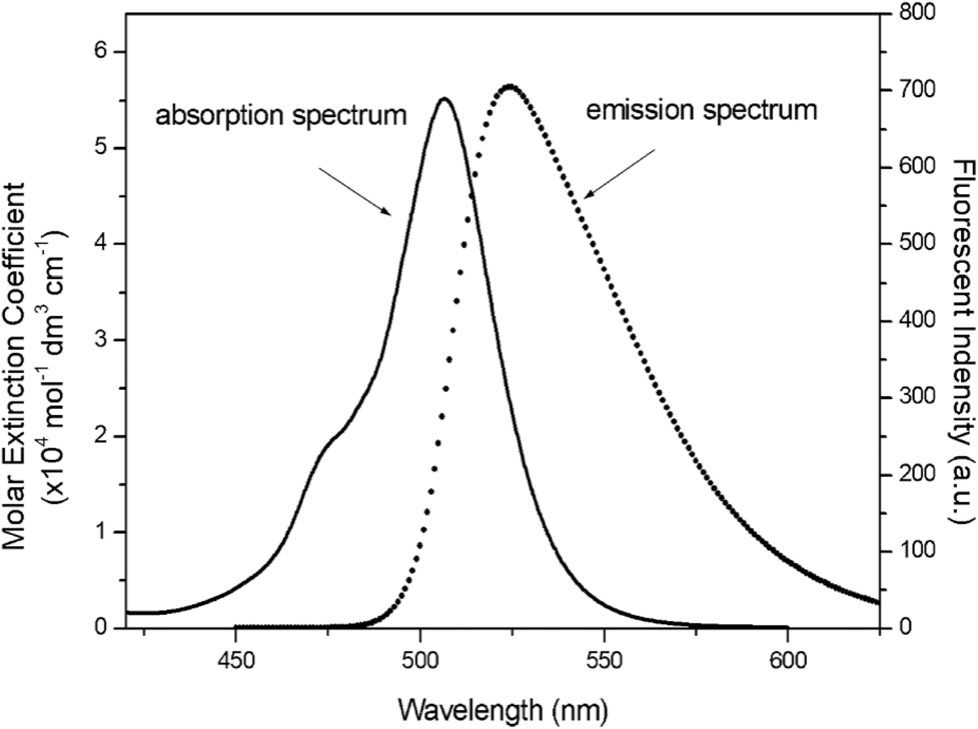
Absorption (solid line) and emission (dotted line) spectra of conjugate 108 in THF. Reproduced with permission from ref. 103. Copyright 2020, Royal Society of Chemistry.

Wang *et al.* reported the synthesis of a *s*-triazine–BODIPY conjugate 107, functionalized with an *N-tert*-butoxycarbonyl protected amine group(–NH_2_). They subsequently prepared conjugate 108*via* deprotection and acetylation of 107 (Scheme 21).^111^ This synthetic route utilized stepwise nucleophilic substitution of cyanuric chloride. Both conjugates exhibited characteristic BODIPY absorption bands centered around 500 nm and emission maxima around 525 nm when measured in acetonitrile solution (Fig. 26).

**Scheme 22:**
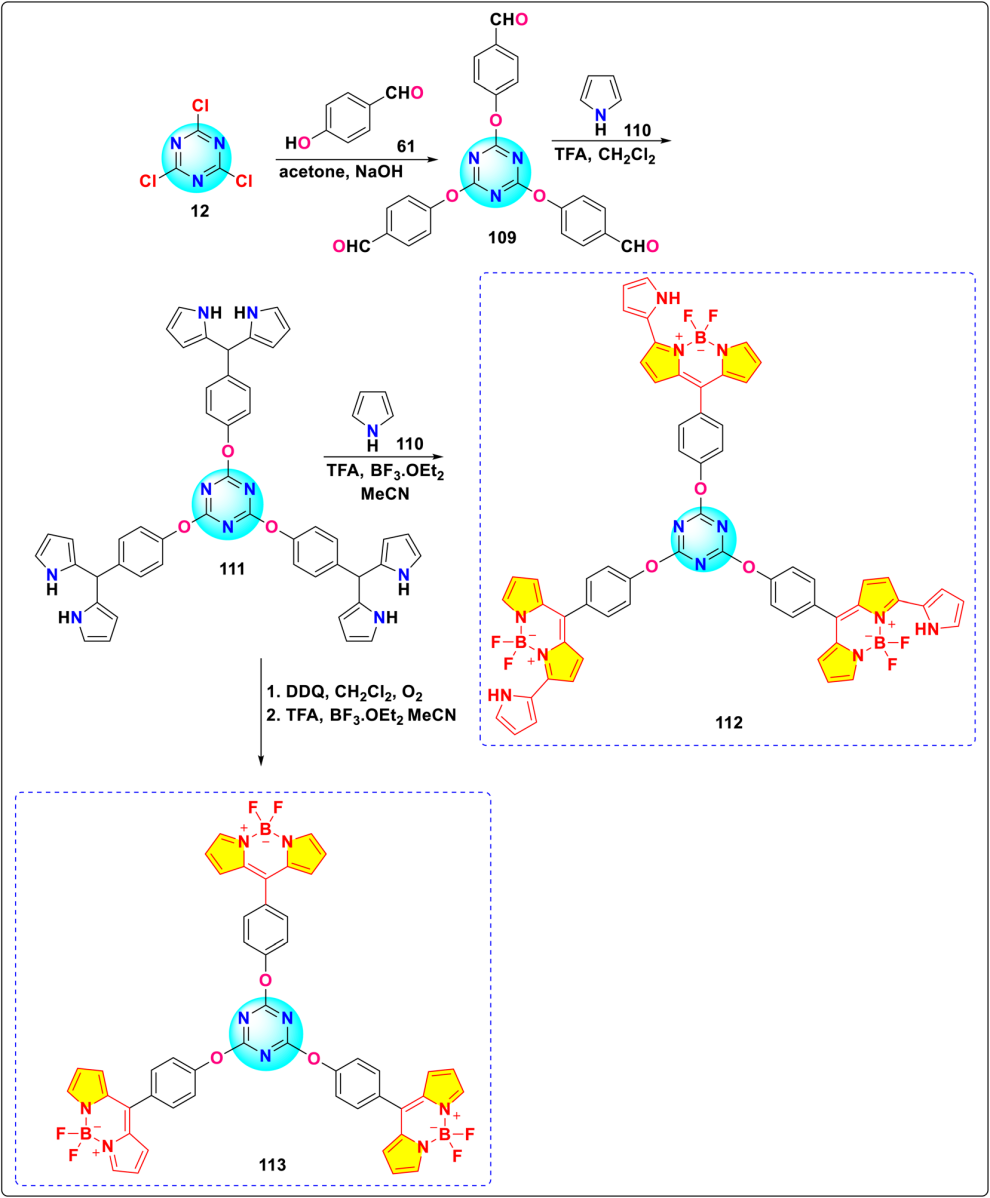
Synthetic route for *s*-triazine-BODIPY conjugates 112 and 113 for molecular viscometer application.

Behera and Ravikanth synthesized two *s*-triazine-BODIPY conjugates, 112 and 113, and compared their properties with a BODIPY monomer (Scheme 22).^112^ Conjugate 112 displayed a hypsochromic shift along with broadening of the absorption spectrum around 507–551 nm compared to its monomer, indicating excitonic coupling between the 3-pyrrolyl BODIPY units. In the emission spectra, the conjugate 112 displayed a blue shift with a lower fluorescence quantum yield than the monomer due to enhanced non-radiative decay pathways. However, conjugate 113 showed absorption and emission spectra similar to those of the BODIPY monomer, indicating almost minimal electronic interaction between the BODIPY units. Conjugate 112 exhibited fluorescence in both solid and liquid states. Viscosity studies revealed that the fluorescence of conjugate 112 increased markedly with solvent viscosity, indicating its promise for assessing cellular viscosity during physiological processes.

**Scheme 23:**
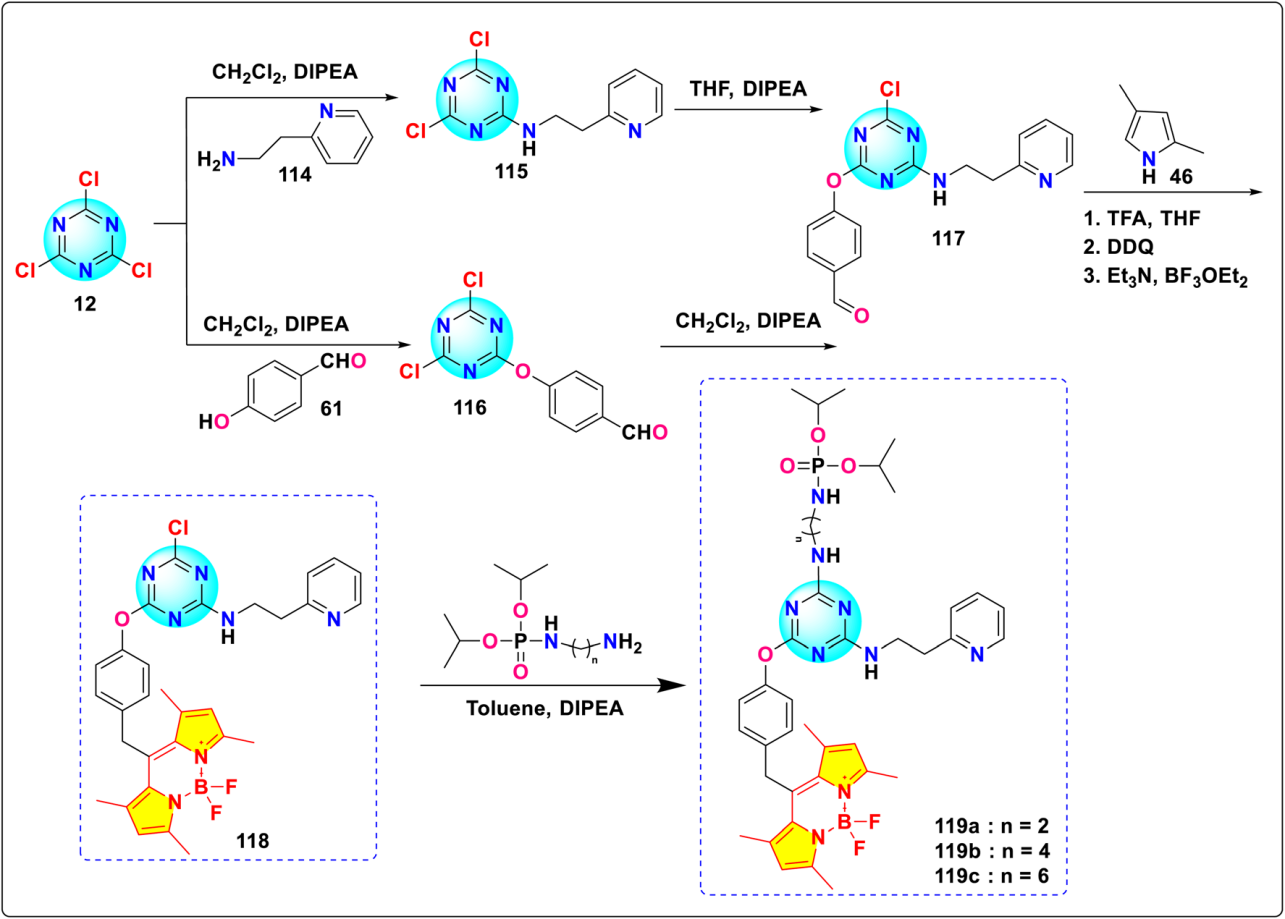
Synthetic route for *s*-triazine-BODIPY conjugates 119a-c.

Marques and colleagues synthesized three novel s-triazine-BODIPY conjugates 119a-c*via* sequential substitution of cyanuric chloride (Scheme 23).^113^ The researchers employed quantum methods and both experimental and computational analysis to evaluate the reaction process and assess the orthogonality of the reaction mechanism. Their investigation revealed the following sequential substitution order based on the ease of nucleophilic displacement: *p*-hydroxybenzaldehyde > 2-(pyridin-2-yl)ethanamine > aminoalkyl phosphoramidate. This detailed understanding of nucleophilic substitution preferences on cyanuric chloride informs the design and synthesis of new fluorophores incorporating similar nucleophilic components.

**Scheme 24:**
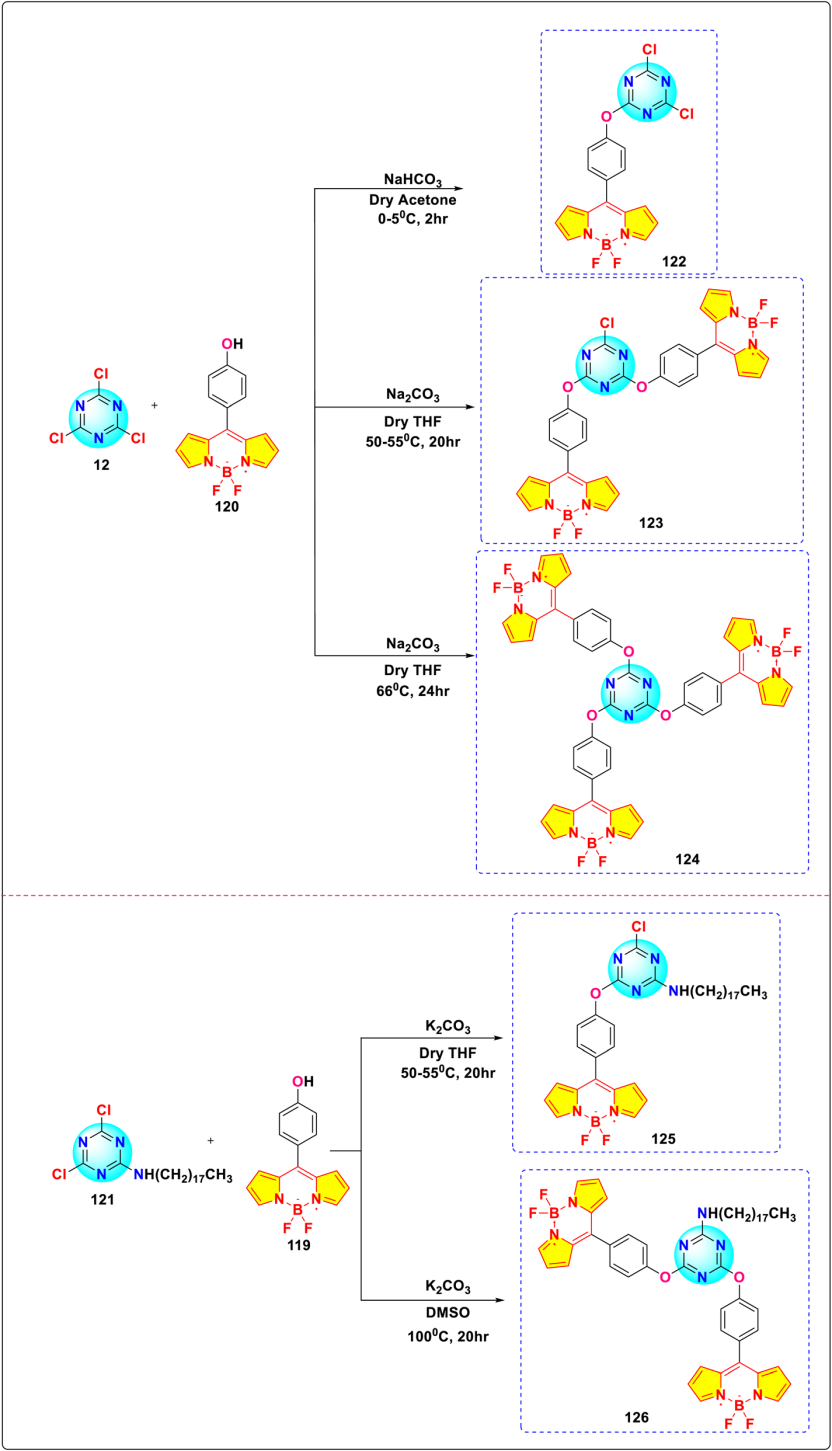
Synthetic route for *s*-triazine-BODIPY conjugates 122-126 for photophysical studies.

**Fig. 27:**
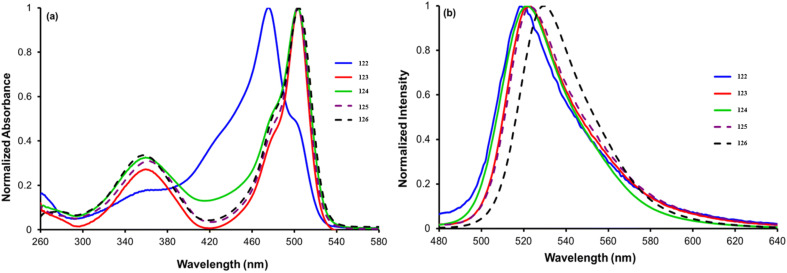
(a) Absorption spectra of conjugates 122-126. (b) Emission spectra of conjugates 122-126. Reproduced with permission from ref. 106. Copyright 2024, Royal Society of Chemistry.

Satardekar and co-workers synthesized a series of *s*-triazine-BODIPY conjugates 122-126 and studied their photophysical behaviour with a focus on their aggregation properties (Scheme 24).^114^ The conjugates displayed a typical BODIPY absorption band around 500 nm due to the S_0_ → S_1_ transition and an additional band around 355 nm due to the S_0_ → S_2_ transition in the DMSO solvent (Fig. 27). The conjugates exhibited a relative fluorescence quantum yield between 0.08 and 0.18, with average fluorescence lifetimes varying from 0.24 ns to 0.11 ns. The aggregation properties of the conjugates were studied in detail and conjugate 125 showed strong aggregation-induced chromic emission with an additional emission peak at 615 nm in 30–50% water/DMSO mixtures, indicating J-aggregate formation.

## 3. Conclusion and outlook

This paper offers a comprehensive summary of the various design principles, synthetic methods, and applications of *s*-triazine BODIPY conjugates over the past decade. It is the first exhaustive review to systematically explore the conjugates' detailed photophysical properties, biological applications, sensing applications, and various other photophysical applications.

Cyanuric chloride serves as a versatile precursor for the controlled introduction of substituents into a symmetric triazine core, facilitated by its sequential, temperature-regulated displacement of chloride groups. This characteristic renders cyanuric chloride the preferred precursor in most synthetic methodologies, wherein substituents are incorporated *via* nucleophilic aromatic substitution (NAS) under condensation reaction conditions. The stepwise reactivity of the three chlorine atoms activated at distinct temperatures enables precise mono-, di-, or tri-substitution of the triazine scaffold. In some cases, instead of direct substitution, cyanuric chloride undergoes initial condensation with propargyl bromide to attach alkyne groups, followed by subsequent derivatization *via* copper-catalyzed azide–alkyne cycloaddition (CuAAC) click chemistry. This approach permits the introduction of diverse substituents under mild, biorthogonal conditions. Finally, in some synthetic approaches, benzonitrile derivatives first undergo reaction with the BODIPY substituents, after which the nitrile groups undergo cyclotrimerization in the presence of trifluoromethanesulfonic acid, yielding *s-*triazine BODIPY conjugates.

The *s*-triazine-BODIPY conjugates have demonstrated significant promise in biological applications, particularly in cancer diagnostics and therapy (Conjugates 5, 11, and 19). Their robust absorption in the visible spectrum and elevated fluorescence quantum yields enable sensitive imaging of live cells. At the same time, heavy-atom–modified derivatives generate singlet oxygen with high efficiency (Conjugate 18), facilitating effective photodynamic therapy against tumour cells.

The conjugates also serve as exceptionally sensitive and selective fluorescent sensors for various chemical species. The tunable photophysical properties of the BODIPY core help to determine the interaction between conjugates and chemical species through the enhancement or quenching of fluorescence emission. In the case of selective detection of biothiols and Cu^2+^ ions, an increase in fluorescence is observed, while sensing Ag^+^ ions results in quenching of fluorescence. These features facilitate practical applications in environmental monitoring of heavy metals and *in vivo* imaging of biologically relevant ions, demonstrating the broad utility of the conjugates as versatile fluorescent chemosensors.

*s*-Triazine-BODIPY conjugates display a remarkable breadth of photophysical functionality. They serve as photocatalysts in artificial photosynthesis as graphene-anchored systems for visible-light NADH regeneration and CO_2_ reduction (*e.g.*, conjugate 38). Furthermore, their incorporation into both dye-sensitized solar cells (DSSCs) and bulk heterojunction organic solar cells (OSCs) contributes significantly to broadening the spectral response and augmenting overall power conversion efficiencies, occasionally in synergistic combination with auxiliary materials such as reduced graphene oxide (*e.g.*, conjugates 42, 90). Beyond energy applications, specific *s*-triazine-BODIPY conjugates function as molecular rotors, sensitively reporting on microenvironmental viscosity changes in various media, including live cells (*e.g.*, conjugates 52a, 86, 112). Others, particularly halogenated derivatives, serve as potent singlet oxygen generators for photochemical applications (*e.g.*, conjugates 54a, 54b). Furthermore, specific conjugates have been engineered into functional materials such as liquid crystals exhibiting nematic or columnar phases (*e.g.*, conjugates 68, 69, 84, 85), components for photodiodes (*e.g.*, conjugates 96, 98), platforms for bioconjugation *via* activated esters (*e.g.*, conjugate 60), and systems demonstrating efficient FRET or AIE phenomena (*e.g.*, conjugates 102, 104, 125).

Despite the demonstrated potential, certain limitations persist; for instance, aggregation-induced quenching (ACQ) or H-aggregation effects can diminish fluorescence intensity and quantum yield in derivatives with multiple BODIPY units, particularly those with high molecular symmetry that promote π–π stacking interactions. Furthermore, while conjugates show promise in photodynamic therapy (PDT) and as photosensitizers, optimizing their efficacy, cellular uptake, and targeted action remains an area for refinement. Similarly, enhancing power conversion efficiencies and long-term stability is crucial for their broader application in solar cell technologies.

The future outlook for *s*-triazine-BODIPY conjugates is bright, driven by their tunable characteristics and the synergistic potential of combining the *s*-triazine and BODIPY scaffolds. There is considerable scope for expanding their biological applications, designing conjugates with enhanced anticancer potency or targeted delivery mechanisms, and exploring their utility beyond cancer therapy. In sensing, efforts should focus on creating highly selective and sensitive probes that can operate in various real-world environments and different pH ranges. Furthermore, the presence of coexisting ions (such as Cu^2+^ and Fe^3+^) and organic compounds in intricate samples can obstruct selectivity, leading to inaccurate signals or fluorescence reduction. Moreover, integrating conjugates into testing papers represents a particularly cost-efficient approach to identify a variety of analytes in practical environments. By tackling these hurdles and leveraging existing expertise, *s*-triazine-BODIPY conjugates possess considerable potential for ongoing advancements across various scientific disciplines.

## Conflicts of interest

The authors declare that they have no known competing financial interests or personal relationships that could have appeared to influence the work reported in this paper.

## Data Availability

No primary research results, software or code have been included and no new data were generated or analysed as part of this review.
